# Role of activating transcription factor 4 in the hepatic response to amino acid depletion by asparaginase

**DOI:** 10.1038/s41598-017-01041-7

**Published:** 2017-04-28

**Authors:** Rana J. T. Al-Baghdadi, Inna A. Nikonorova, Emily T. Mirek, Yongping Wang, Jinhee Park, William J. Belden, Ronald C. Wek, Tracy G. Anthony

**Affiliations:** 1 Endocrinology and Animal Biosciences Graduate Program, Rutgers, The State University of New Jersey, New Brunswick, NJ 0890 USA; 2 Department of Nutritional Sciences and the New Jersey Institute for Food, Nutrition and Health, Rutgers, The State University of New Jersey, New Brunswick, NJ 08901 USA; 3 Department of Animal Sciences, Rutgers, The State University of New Jersey, New Brunswick, NJ 0890 USA; 4 Department of Biochemistry and Molecular Biology, Indiana University School of Medicine, Indianapolis, IN 46202 USA; 5 Department of Physiology and Pharmacology, College of Veterinary Medicine, University of Al-Qadisiyah, Al-Qadisiayah, Iraq

## Abstract

The anti-leukemic agent asparaginase activates the integrated stress response (ISR) kinase GCN2 and inhibits signaling *via* mechanistic target of rapamycin complex 1 (mTORC1). The study objective was to investigate the protective role of activating transcription factor 4 (ATF4) in controlling the hepatic transcriptome and mediating GCN2-mTORC1 signaling during asparaginase. We compared global gene expression patterns in livers from wildtype, *Gcn2* ^−/−^, and *Atf4* ^−/−^ mice treated with asparaginase or excipient and further explored selected responses in livers from *Atf4* ^+/−^ mice. Here, we show that ATF4 controls a hepatic gene expression profile that overlaps with GCN2 but is not required for downregulation of mTORC1 during asparaginase. Ingenuity pathway analysis indicates GCN2 independently influences inflammation-mediated hepatic processes whereas ATF4 uniquely associates with cholesterol metabolism and endoplasmic reticulum (ER) stress. Livers from *Atf4* ^−/−^ or *Atf4* ^+/−^ mice displayed an amplification of the amino acid response and ER stress response transcriptional signatures. In contrast, reduction in hepatic mTORC1 signaling was retained in *Atf4* ^−/−^ mice treated with asparaginase. *Conclusions*: GCN2 and ATF4 serve complementary roles in the hepatic response to asparaginase. GCN2 functions to limit inflammation and mTORC1 signaling whereas ATF4 serves to limit the amino acid response and prevent ER stress during amino acid depletion by asparaginase.

## Introduction

Asparaginase (ASNase) is a chemotherapy agent used to treat acute lymphoblastic leukemia, the most common childhood cancer^1^. Asparaginase induces remission by depleting blood levels of asparagine and glutamine, starving leukemic lymphoblasts of substrates essential for tumor growth^2, 3^. In liver and other tissues, amino acid depletion by asparaginase is sensed by general control nonderepressible 2 (GCN2 or EIF2AK4), a kinase which phosphorylates eukaryotic initiation factor 2 on its alpha subunit^4–6^. Phosphorylation of eIF2 slows the delivery of initiator tRNA to the translation machinery, altering gene-specific translation and promoting synthesis of basic-region leucine zipper DNA binding proteins such as Activating Transcription Factor 4 (ATF4 or CREB2)^7^. Binding of ATF4 to CAAT/enhancer binding protein-ATF response elements in DNA upon amino acid deprivation activates a homeostatic transcriptional program called the amino acid response (AAR) which regulates nutrient uptake and metabolism, energy and redox homeostasis, and cell cycle control^8, 9^. Animals lacking GCN2 fail to increase eIF2 phosphorylation and induce the AAR in response to asparaginase, and these failures correspond with hepatic inflammation and toxicity, pancreatitis, and immunosuppression leading to terminal morbidity^6, 10–12^. To what extent these outcomes are attributed to a lack of ATF4 induction is unknown.

A family of eIF2 kinases instigates eIF2 phosphorylation under diverse stress conditions. The integration of this information at the level of eIF2 is described as the integrated stress response (ISR)^13^ and results in ATF4 synthesis. Upon endoplasmic reticulum stress, phosphorylation of eIF2 by protein kinase R-like ER-resident (PERK or EIF2AK3) leads to ATF4 synthesis and in combination with the actions of activating transcription factor 6 (ATF6) and inositol requiring element 1 reduces the client load and increases chaperone mediated recovery processes as part of the unfolded protein response (UPR)^14–17^. It is suggested that during ER stress, the cellular choice between adaptation versus apoptosis depends on the extent and duration of ATF4 expression and its target gene C/EBP Homologous Protein (CHOP or DDIT3/GADD153)^18, 19^. While the canonical ISR positions ATF4 at a key point in tailoring the resulting transcriptional signatures to ER stress^18, 20^, little is known concerning the global contribution of ATF4 to guiding the transcriptional AAR following amino acid depletion *in vivo*.

Changes in amino acid sufficiency are sensed by mammalian target of rapamycin complex 1 (mTORC1), a regulator complex located at the lysosomal surface^21^. Phosphorylation of mTORC1 and its substrates, ribosomal protein S6 kinase (S6K1) and eIF4E-binding protein 1 (4E-BP1), drive changes in hepatic translational efficiency and ribosomal capacity in response to dietary amino acid supply^22^. Amino acid deficiency *via* asparaginase treatment also inhibits hepatic mTORC1 signaling; however, mTORC1 is activated in the livers of *Gcn2* ^−/−^ mice, leading to hyperphosphorylation of S6K1 and 4E-BP1^5^. Several studies suggest that transcriptional induction of Sestrin2 by ATF4 coordinates mTORC1 inhibition during nutrient deprivation^23–25^. Thus, repression of mTORC1 during asparaginase treatment may also be under the control of ATF4.

Previous studies by our lab and others indicate that GCN2 serves to protect the liver during asparaginase treatment^6, 10–12^ and chronic liver injury by carbon tetrachloride^26^. Based on this scientific premise, our objective was to determine the role of ATF4 in mediating hepatic AAR activation, reduced mTORC1 signaling and liver protection during asparaginase. To address this objective, we administered asparaginase to mice lacking *Gcn2* or *Atf4* and performed RNA sequencing (RNA-Seq) to measure global gene expression changes caused by the treatment. Ingenuity pathway analysis of the differential hepatic gene expression revealed that GCN2 plays a major role in inflammation-mediated regulation of hepatic processes whereas ATF4 regulates cholesterol metabolism and signaling via eIF2, eIF4 and ER stress. Together the GCN2-ATF4 axis was implicated in cytochrome P450-mediated reactions and cytoplasmic nuclear receptor activation. Selected hepatic responses to asparaginase were further evaluated in these mice plus those with heterozygous *Atf4* deletion to assess haploinsufficiency. These experiments further showed that 1) total or partial loss of ATF4 amplifies the AAR during chronic amino acid depletion, leading to induction of ER stress, 2) GCN2, but not ATF4, influences mTORC1 activity during amino acid depletion by asparaginase and 3) asparaginase-induced increases in Sestrin2 expression and phosphorylation do not require GCN2 or ATF4. These findings substantially advance our understanding of the role of GCN2 versus ATF4 in the hepatoprotective responses to amino acid depletion and provide new insight into the mechanisms by which asparaginase causes adverse metabolic events.

## Results

### Whole body responses to asparaginase in *Atf4*^−*/*−^ mice differ from *Gcn2*^−*/*−^ mice

**Figure 1:**
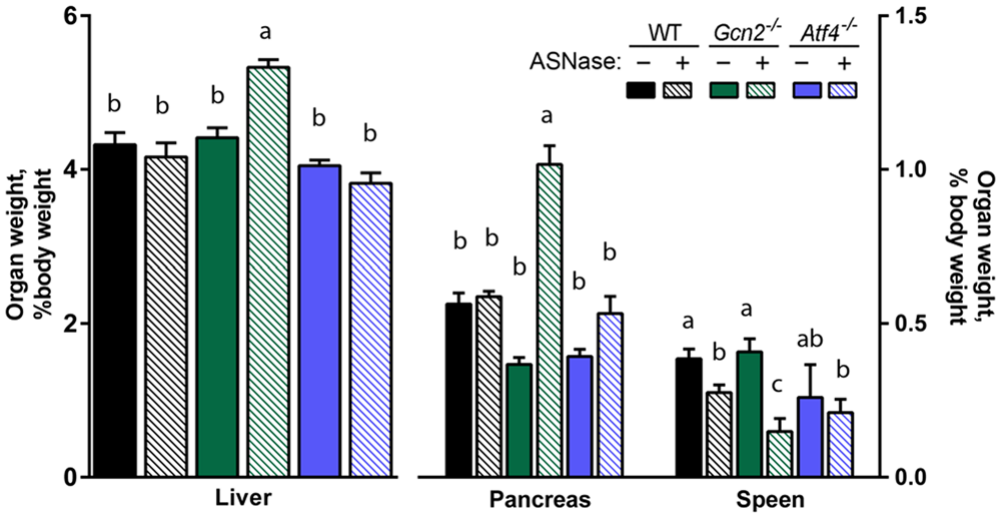
Percent weight change of liver, pancreas, and spleen relative to body weight following 8 daily injections of asparaginase (3 IU per gram body weight, ASNase) or phosphate buffered saline excipient (PBS) in wild type mice (WT) or mice deleted for *Gcn2* (*Gcn2* ^−/−^) or *Atf4* (*Atf4* ^−/−^). Data are represented as the average value ± standard error of the mean, n = 4–6 animals per group. Means not sharing a letter are different according to Tukey post hoc analysis following ANOVA, P < 0.05.

We sought to understand the role of ATF4 relative to GCN2 in hepatoprotective responses to asparaginase treatment. To accomplish this and to capture both the acute as well as the auxiliary stress responses to asparaginase, we injected asparaginase (3 IU/g) into WT, *Gcn2* ^−/−^, and *Atf4* ^−/−^ mice once daily for 8 d using phosphate buffered saline (PBS) as a control. We chose this time point based on our previous studies which show that a single injection of asparaginase alters hepatic signaling and transcriptional responses in WT and *Gcn2* ^−/−^ mice similar to multiple injections^5, 10, 11^. Before treatment commenced, *Atf4* ^−/−^ mice had significantly less fat mass than WT and *Gcn2* ^−/−^ mice (Supplementary Fig. S1), in agreement with the observation that *Atf4* ^−/−^ mice are leaner than *Atf4* ^+/+^ mice^27^. Following asparaginase treatment, WT mice experienced minimal change in body weight and body composition, but *Atf4* ^−/−^ and *Gcn2* ^−/−^ mice both lost body weight and body fat (Supplementary Fig. S1). Compared to WT mice, asparaginase increased liver and pancreas mass and reduced spleen mass in *Gcn2* ^−/−^ mice (Fig. 1) in accordance with our previous findings^6, 10, 12, 27^. None of these asparaginase-associated effects in *Gcn2* ^−/−^ mice were observed in *Atf4* ^−/−^ mice; instead, *Atf4* ^−/−^ organ/tissue masses did not significantly change following asparaginase treatment.

### ATF4 and GCN2 control overlapping patterns of hepatic gene expression in response to asparaginase

To further define the role of ATF4 and GCN2 in liver in response to asparaginase, we performed transcriptional profiling of poly-A mRNA via massively parallel sequencing of cDNA (RNA-seq). To accomplish this, total RNA was extracted from liver of WT, *Gcn2* ^−/−^ and *Atf4* ^−/−^ mice treated with asparaginase or PBS excipient. After RNA-Seq, we examined data quality by examining the percentages of mapped reads and evaluated fragments per kilobase of transcript per million mapped reads (FPKM) values for isoforms, transcripts and annotated genes (Supplementary Table S1). Next, we confirmed the *Gcn2* ^−/−^ and *Atf4* ^−/−^ deletions at the genome level (Supplementary Fig. S1). We also assessed the degree of global changes in gene expression among the samples visualized as cross-comparison Volcano plots (Supplementary Fig. S3). Furthermore, Principle Component Analysis indicated that transcriptional profiles from the livers of asparaginase-treated *Atf4* ^−/−^ and *Gcn2* ^−/−^ mice were quite different from the other treatment groups (Supplementary Fig. S3).

**Figure 2:**
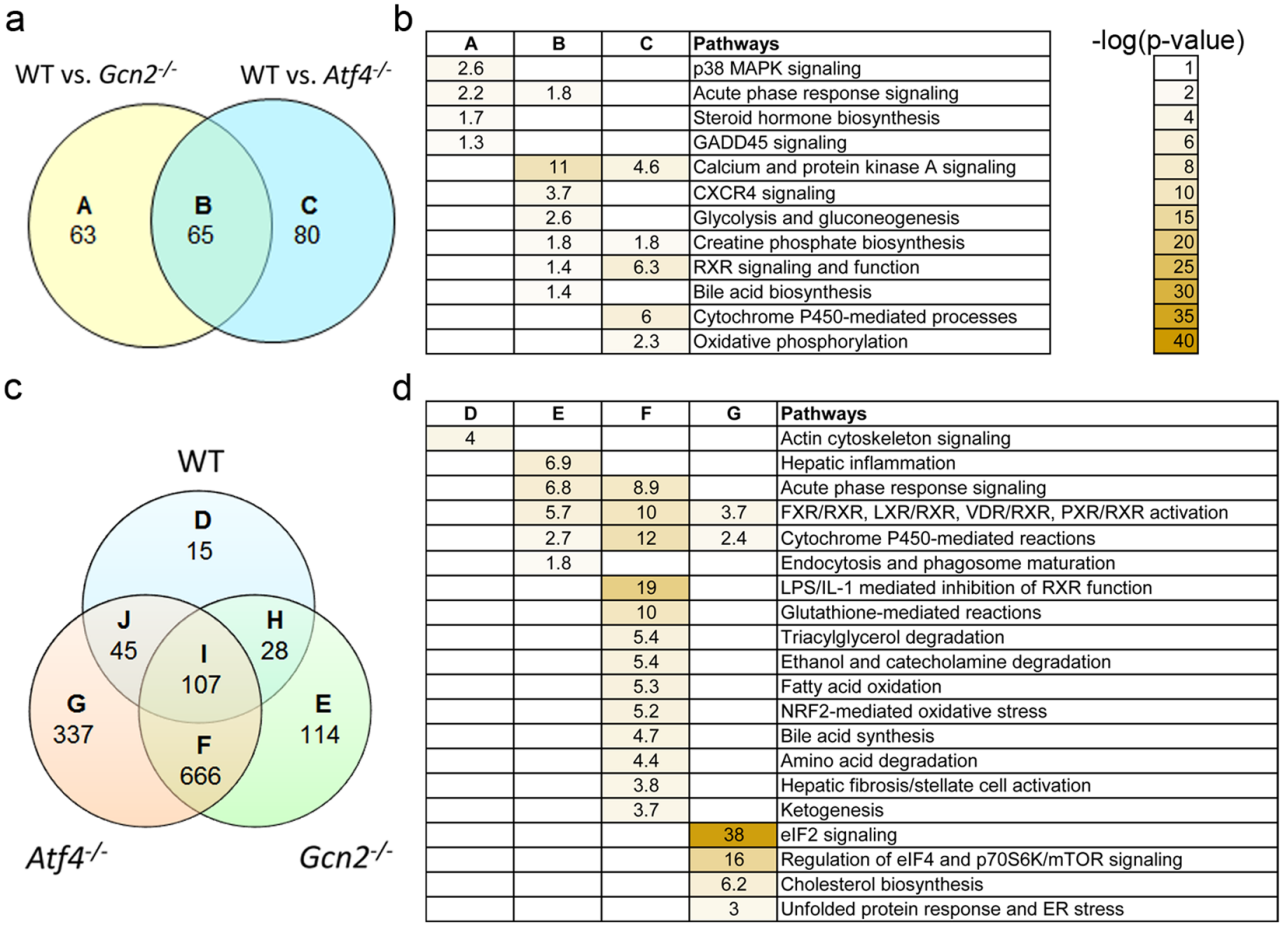
Transcriptional profiling of livers following 8 daily injections of asparaginase (3 IU per gram body weight, ASNase) or phosphate buffered saline excipient (PBS) in wild type mice (WT) or mice deleted for *Gcn2* (*Gcn2* ^−/−^) or *Atf4* (*Atf4* ^−/−^). (**a**) Venn diagram shows the number of hepatic genes altered basally, categorized as unique to *Gcn2* ^−/−^ [A], unique to *Atf4* ^−/−^ [C] or common to deletion of either [B]. (**b**) Overview of the top overrepresented canonical pathways based on gene expression changes in panel a. (**c**) Venn diagram shows ASNase alters the transcriptome differently in WT, *Gcn2* ^−/−^ and *Atf4* ^−/−^ mice, with gene changes clustered as unique to WT [D], unique to *Gcn2* ^−/−^ [E]; unique to *Atf4* ^−/−^ [G]; common to *Gcn2* ^−/−^ and *Atf4* ^−/−^ [F]; common to WT and *Gcn2* ^−/−^ [H]; common to WT and *Atf4* ^−/−^ [J]; and common to all strains [I]. (**d**) Overview of the top overrepresented canonical pathways based on gene expression changes in panel c. Data represent n = 3 per group. All differentially expressed genes were statistically significant at a false discovery rate (q value) < 0.1.

A comparative examination of PBS-injected mice by Venn analysis showed many hepatic genes required *Gcn2* and/or *Atf4* for basal expression (Fig. 2a; Supplementary Table S2). We found 128 genes were expressed differently upon *Gcn2* deletion whereas deletion of *Atf4* changed basal expression of 145 genes compared to WT. Among these, 65 genes were altered in both *Gcn2* ^−/−^ and *Atf4* ^−/−^ mice (category B). Ingenuity pathway analyses of these gene categories (Fig. 2b; Supplementary Table S3) indicated that loss of either *Gcn2* or *Atf4* resulted in basal changes in the expression of genes involved in calcium and protein kinase A signaling, chemokine receptor 4 (CXCR4) signaling, glycolysis and gluconeogenesis, creatine phosphate biosynthesis, retinoid acid receptor (RXR) signaling and function, bile acid biosynthesis and acute phase response signaling. Loss of GCN2 in basal state uniquely affected p38 MAPK signaling, steroid hormone biosynthesis, and growth arrest and DNA damage 45 (GADD45) signaling in liver. Loss of ATF4 in basal state uniquely affected cytochrome P450-mediated processes and oxidative phosphorylation. Thus, while the global influence of GCN2 in the non-stressed liver was largely mediated by ATF4, a few key biological processes were unique to each protein. These findings indicate that both GCN2 and ATF4 function to regulate gene expression in the absence of apparent stress and these key components of the ISR serve complementary roles in regulating liver metabolism and signaling.

We then utilized Venn analysis to identify shared versus unique hepatic gene patterns altered in *Gcn2* ^−/−^ and *Atf4* ^−/−^ mice treated with asparaginase (ASNase versus PBS in each genetic strain) (Fig. 2c, Supplementary Table S2). In WT mice, asparaginase altered expression of 195 genes compared to PBS. Consistent with our Principal Component Analysis, deficiency in either *Gcn2* or *Atf4* promoted much larger asparaginase-induced shifts in gene expression, reflected by increased numbers of genes in *Gcn2* ^−/−^ (915 genes) and *Atf4* ^−/−^ (1,155 genes) categories. Roughly one-half of these gene expression changes were common to loss of *Gcn2* and *Atf4* but not shared with WT (category F). Among the genes altered by asparaginase, 114 genes in *Gcn2* ^−/−^ were not shared with either *Atf4* ^−/−^ or WT (category E) whereas 337 genes altered in *Atf4* ^−/−^ mice were not shared with *Gcn2* ^−/−^ or WT (category G). Ingenuity pathway analysis of these specified gene categories (Fig. 2d; Supplementary Table S3) identified hepatic inflammation, endocytosis and phagosome maturation as altered by *Gcn2* status (category E) whereas loss of *Atf4* influenced signaling via eIF2, eIF4, p70S6K/mTOR, ER stress and the UPR as well as cholesterol biosynthesis (category G). We also found that a variety of metabolic processes were misaligned in both *Gcn2* ^−/−^ and *Atf4* ^−/−^ mice (category F). For example, genes implicated in cytoplasmic nuclear receptor activation, glutathione-mediated reactions, nuclear factor 2 (NRF2)-mediated oxidative stress, triglyceride, ethanol and amino acid degradation, fatty acid oxidation, acute phase response, cytochrome P450-mediated reactions, bile acid synthesis, and hepatic fibrosis were altered in both *Gcn2* ^−/−^ and *Atf4* ^−/−^ mice. Additional examination of shared gene categories by heat map showed gene expression changes in *Gcn2* ^−/−^ and *Atf4* ^−/−^ in category F were largely in the same direction whereas many gene expression changes in category I were not shared in direction (Supplementary Fig. S4).

Among the biological processes identified, cytochrome P450-mediated reactions and activation of cytoplasmic nuclear receptors (e.g., FXR, RXR, PXR, LXR, VDR) stood out as shared among categories E, F, G. Interestingly, biomolecular data mining using EVEX (evexdb.org) indicated that ATF4 binds LXR, PXR and RXR and coregulates multiple gene families; many of these encode transcriptional and translational responses to ER and oxidative stress in addition to the metabolism of xenobiotics, lipids, cholesterol and bile acids^28, 29^. The directional change in the expression of genes involved in these two biological processes was for the most part shared, reflecting downregulation during asparaginase exposure when the ISR is disrupted.

### Loss of *Atf4* versus *Gcn2* differentially regulates the amino acid response to asparaginase

**Figure 3:**
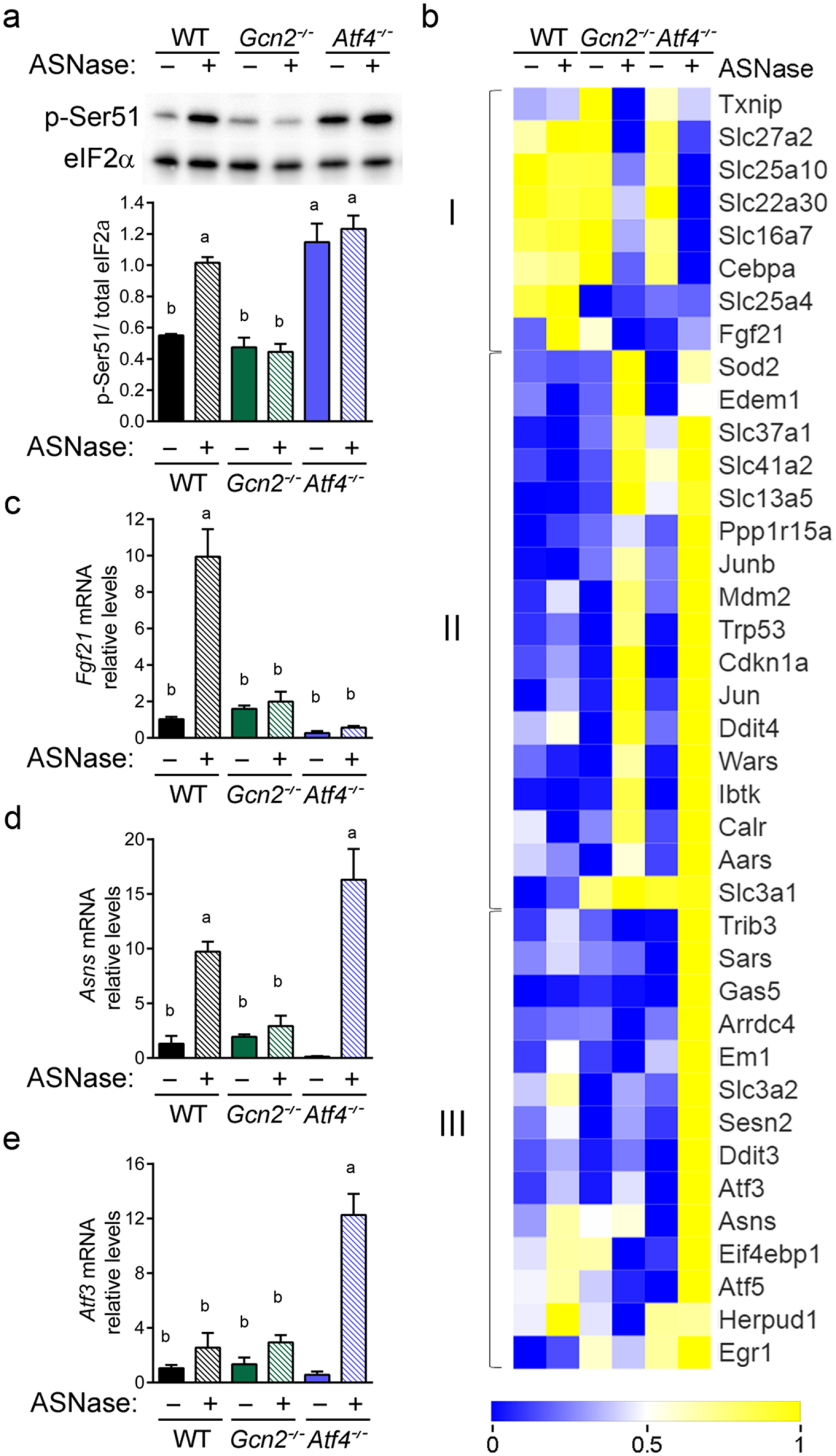
Changes in the amino acid response following 8 daily injections of asparaginase (3 IU per gram body weight, ASNase) or phosphate buffered saline excipient (PBS) in wild type mice (WT) or mice deleted for *Gcn2* (*Gcn2* ^−/−^) or *Atf4* (*Atf4* ^−/−^). (**a**) Phosphorylation of eIF2 alpha at serine 51 in liver by immunoblot analysis. A full immunoblot is presented in Supplementary Fig. S7. (**b**) Clustered RNA-Seq expression data (n = 3 per group) presented in a heat map illustrates relative mean change in amino acid stress response genes^30^ to ASNase in WT, *Gcn2* ^−/−^ and *Atf4* ^−/−^ mice. Color scale reflects increased (yellow) and decreased (blue) expression of each gene relative across treatment groups. (**c**) Gene expression of *Fgf21* (**d**) *Asns* and (**e**) *Atf3* in liver as measured by RT-qPCR. Data in (**a**,**c**,**d**,**e**) are represented as the average value ± standard error of the mean, n = 3–4 per group. Means without a common letter are different according to Tukey post hoc analysis following ANOVA, P < 0.05.

GCN2 is critical for activation of the ISR by asparaginase in liver but to what extent its downstream effector ATF4 in the implementation of the resulting AAR transcriptional signature is undefined. To address this question, we first measured phosphorylation of eIF2 alpha (p-eIF2) and found that in contrast to loss of *Gcn2*, loss of *Atf4* amplified basal p-eIF2 similar to levels induced by asparaginase (Fig. 3a). To visualize how these differences in phosphorylation impacted the amino acid response we crafted a z-score heat map of selected hepatic genes, with the selection list based on a previously published mRNA expression profile in liver cells subjected to amino acid deprivation^30^ (Fig. 3b). Cluster analysis of these data again revealed shared and unique response patterns upon asparaginase treatment: reduced AAR gene levels in both *Gcn2* ^−/−^ and *Atf4* ^−/−^ (I), increased AAR gene levels in both *Gcn2* ^−/−^ and *Atf4* ^−/−^ (II) and increased AAR gene levels in *Atf4* ^−/−^ but not *Gcn2* ^−/−^ (III). We used RT-qPCR to further assess specific gene transcripts and confirmed that loss of either *Gcn2* ^−/−^ or *Atf4* ^−/−^ altered *Fgf21* mRNA expression to asparaginase in a similar direction (Fig. 3c) whereas *Asns*, *Atf3, Eif4ebp1*, and *Sestrin2* were differentially altered (Fig. 3d,e; Supplementary Fig. S5). These analyses indicate that overall, loss of *Gcn2* dampens the AAR whereas loss of *Atf4* amplifies the AAR following asparaginase exposure.

### Deletion of *Atf4* promotes ER stress to asparaginase

**Figure 4:**
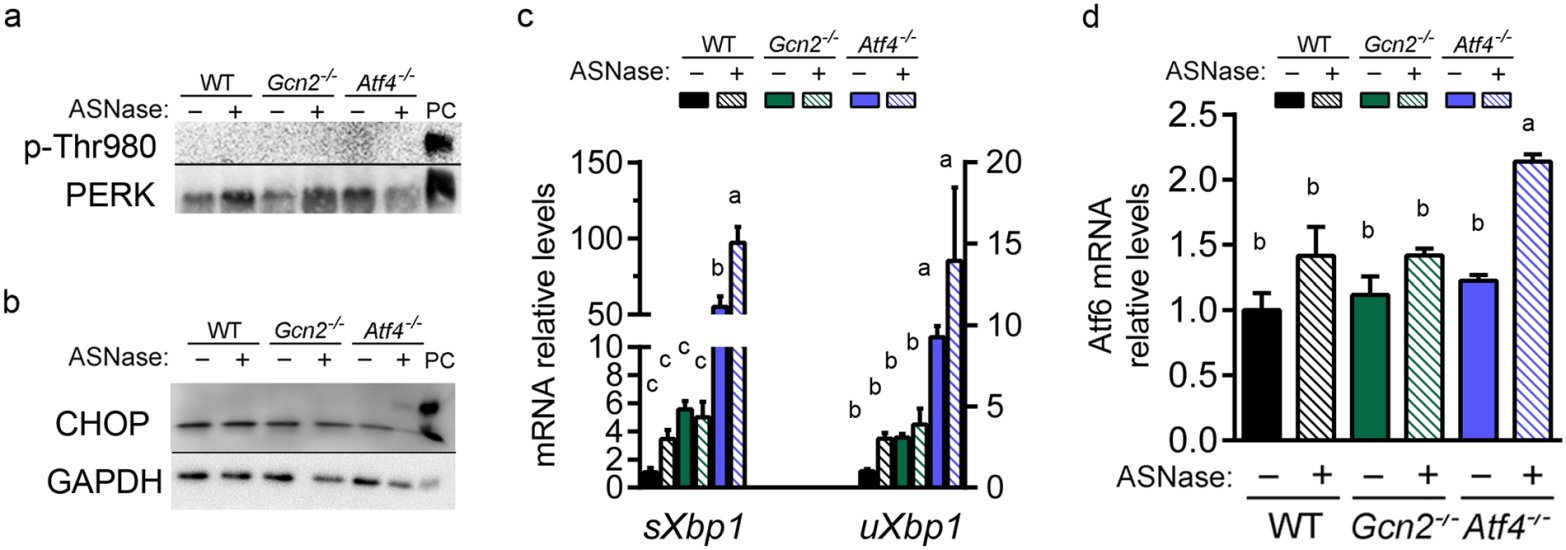
*Atf4* deletion selectively engages the UPR following 8 daily injections of asparaginase (3 IU per gram body weight, ASNase) or phosphate buffered saline excipient (PBS) in wild type mice (WT) or mice deleted for *Gcn2* (*Gcn2* ^−/−^) or *Atf4* (*Atf4* ^−/−^). (**a**) Phosphorylation of PERK in liver by immunoblot analysis. (**b**) CHOP protein expression by immunoblot analysis. (**c**) Gene expression of spliced (s*Xbp1*) and unspliced (u*Xbp1*) *Xbp1* mRNA measured by RT-qPCR. (**d**) *Atf6* mRNA levels measured by RT-qPCR. Data are represented as the average value ± standard error of the mean, n = 3–4 per group. Means without a common letter are different according to Tukey post hoc analysis following ANOVA, P < 0.05. PC, liver from a tunicamycin-treated mouse as positive control. Full immunoblots are presented in Supplementary Figure S7.

Amplification of the hepatic AAR in asparaginase-treated *Atf4* ^−/−^ mice and the pathway analysis revealed that components of the ER stress pathway were also highly enriched. To test this premise further, we assessed the phosphorylation status of PERK by immunoblot, examining electrophoretic migration of PERK protein and measuring signal intensity following incubation with an antibody that specifically recognizes PERK phosphorylated at threonine 980. Both immunoblots showed that PERK phosphorylation was not detectable in any samples, only the positive control (Fig. 4a). However, CHOP protein was detectible in the liver of asparaginase-treated *Atf4* ^−/−^ mice (Fig. 4b). Also, an important measure of ER stress, *Xbp1* mRNA splicing, was greatly elevated in livers from asparaginase-treated *Atf4* ^−/−^ mice (Fig. 4c) as was *Atf6* mRNA expression (Fig. 4d). These results indicate that loss of ATF4 function during sustained amino acid depletion results in selective engagement of the UPR upon asparaginase treatment by a mechanism that does not require PERK phosphorylation.

### GCN2 regulates mTORC1 independent of ATF4

**Figure 5:**
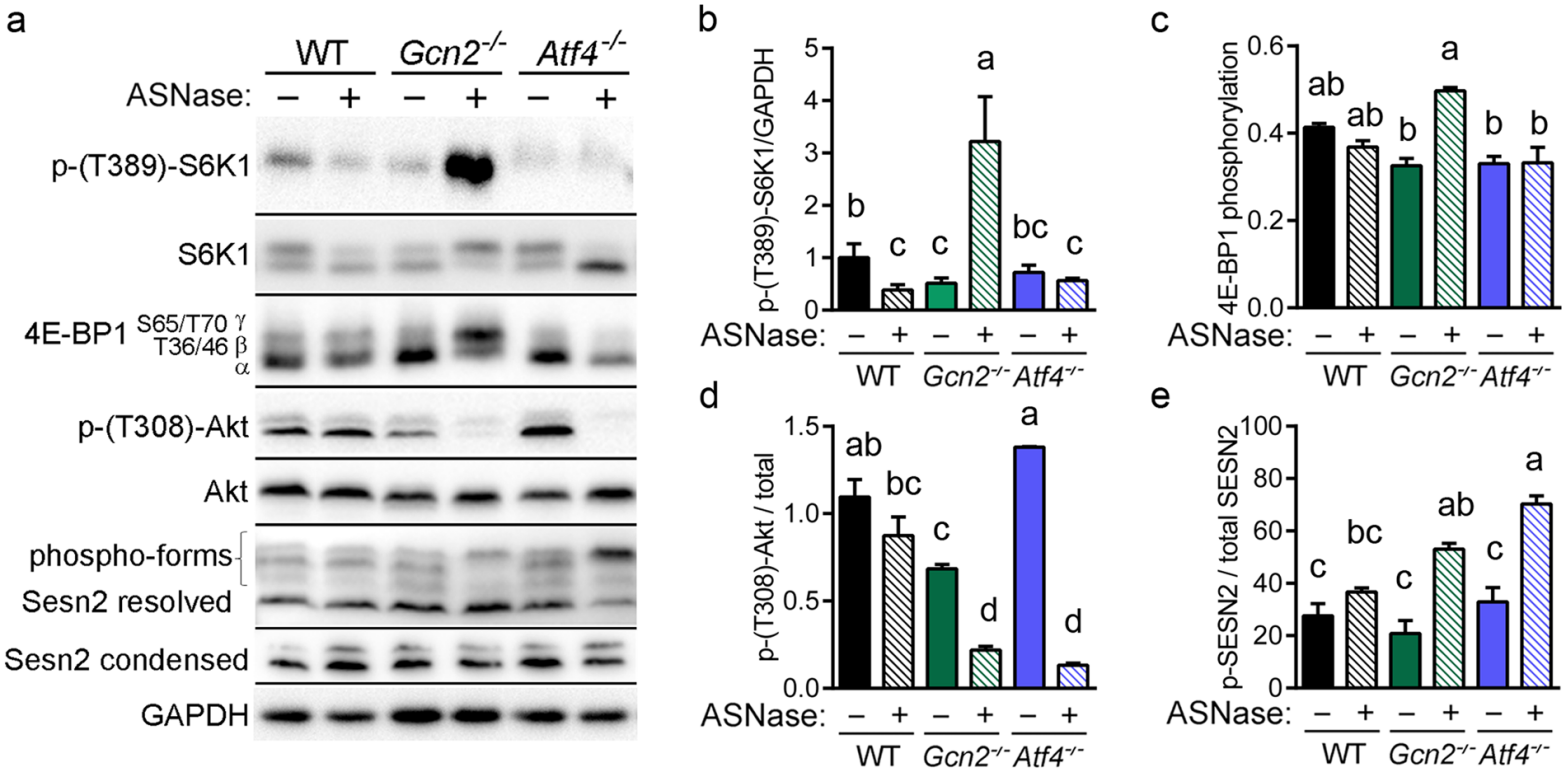
The mTORC1 pathway is amplified in livers from *Gcn2* ^−/−^ but not *Atf4* ^−/−^ mice following 8 daily injections of asparaginase (3 IU per gram body weight, ASNase). (**a**) Representative immunoblots for panels (b–e). (**b**) The ratio of phospho-S6K1 at threonine 389 to glyceraldehyde 3-phosphate dehydrogenase (GAPDH). (**c**) Phosphorylation of 4E-BP1 expressed as the ratio of the γ-form to the α + β + γ sum. (**d**) The ratio of phospho Akt at threonine 308 to total Akt. (**e**) The ratio of Sestrin2 phospho-forms to the sum of all Sesn2 resolved forms. Data are represented as the average value ± standard error of the mean, n = 4–6 per group. Means without a common letter are different according to Tukey post hoc analysis following ANOVA, P < 0.05. Full immunoblots are presented in Supplementary Fig. S7.

Mice lacking *Gcn2* show hyperactivation of mTORC1 in liver during asparaginase^5, 11^ and during dietary leucine deprivation^31, 32^. To explore the connection among GCN2, ATF4, and mTORC1 in response to asparaginase, we first examined the phosphorylation state of S6K1 and 4E-BP1, two readouts of mTORC1 activity. Consistent with our previously published findings, phosphorylation levels of S6K1 and 4E-BP1 in liver were stable or even reduced by asparaginase in WT but amplified in *Gcn2* ^−/−^ mice (Fig. 5a–c). One idea to explain this disruption involves GCN2 regulation of mTORC1 via ATF4^23^. In contrast with this idea, mTORC1 signaling was repressed in *Atf4* ^−/−^ similar to WT. Further, *Ei4ebp1* hepatic gene expression was increased by asparaginase in WT and *Atf4* ^−/−^ mice but not *Gcn2* ^−/−^ mice (Supplementary Fig. S5). To address the underlying mechanism, we measured phosphorylation of protein kinase B/Akt (p-Thr308) to determine if hyperactivated mTORC1 was due to enhanced insulin/growth factor signaling in the *Gcn2* ^−/−^ condition (Fig. 5a,d). However, Akt phosphorylation levels were unchanged in WT and reduced in both *Gcn2* ^−/−^ and *Atf4* ^−/−^, indicating that differences in mTORC1 activity were not due to increased growth factor signaling via Akt. We also examined Sestrin2 because increases in expression and phosphorylation inversely correlate with mTORC1 activity during leucine deprivation^25, 33^. In contrast with this idea, asparaginase increased *Sestrin2* mRNA expression and protein phosphorylation in all mouse strains, with *Atf4* ^−/−^ mice demonstrating the largest increases (Supplementary Fig. S5; Fig. 5a,e). These results indicate that GCN2 but not Sestrin2 or ATF4 are required for hepatic mTORC1 inhibition during sustained amino acid depletion by asparaginase.

### Heterozygous loss of *Atf4* promotes hepatic ER stress and apoptosis to asparaginase

*Atf4* ^−/−^ mice exhibit pleiotropic phenotypes of reduced growth, lack of vision, and defects in hematopoiesis and glucose homeostasis^27, 34–36^. These health challenges may serve as confounders in the assessment of *Atf4* deletion during asparaginase exposure. To help assess this, we decided to examine the impact of asparaginase exposure in Atf4 heterozygotes (*Atf4* ^+/−^) which appear normal and healthy. To understand if ATF4 haploinsufficiency altered the hepatic ISR and health status to asparaginase, we examined the effects of asparaginase on *Atf4* ^+/−^ mice compared to WT and *Atf4* ^−/−^. Prior to treatment, *Atf4* ^−/−^ but not *Atf4* ^+/−^ mice were leaner than *Atf4* ^+/+^ mice (Supplementary Fig. S6). Asparaginase reduced body weight in all strains, with *Atf4* ^+/−^ mice displaying body weights that were intermediate between *Atf4* ^+/+^ and *Atf4* ^−/−^ mice (Supplementary Fig. S6). Asparaginase did not significantly alter liver or pancreas mass but reduced spleen weight in all strains (Supplementary Fig. S6). Asparaginase also elevated liver triglycerides in all strains, although to a lesser extent in *Atf4* ^−/−^ mice (Supporting Fig. S6).

**Figure 6:**
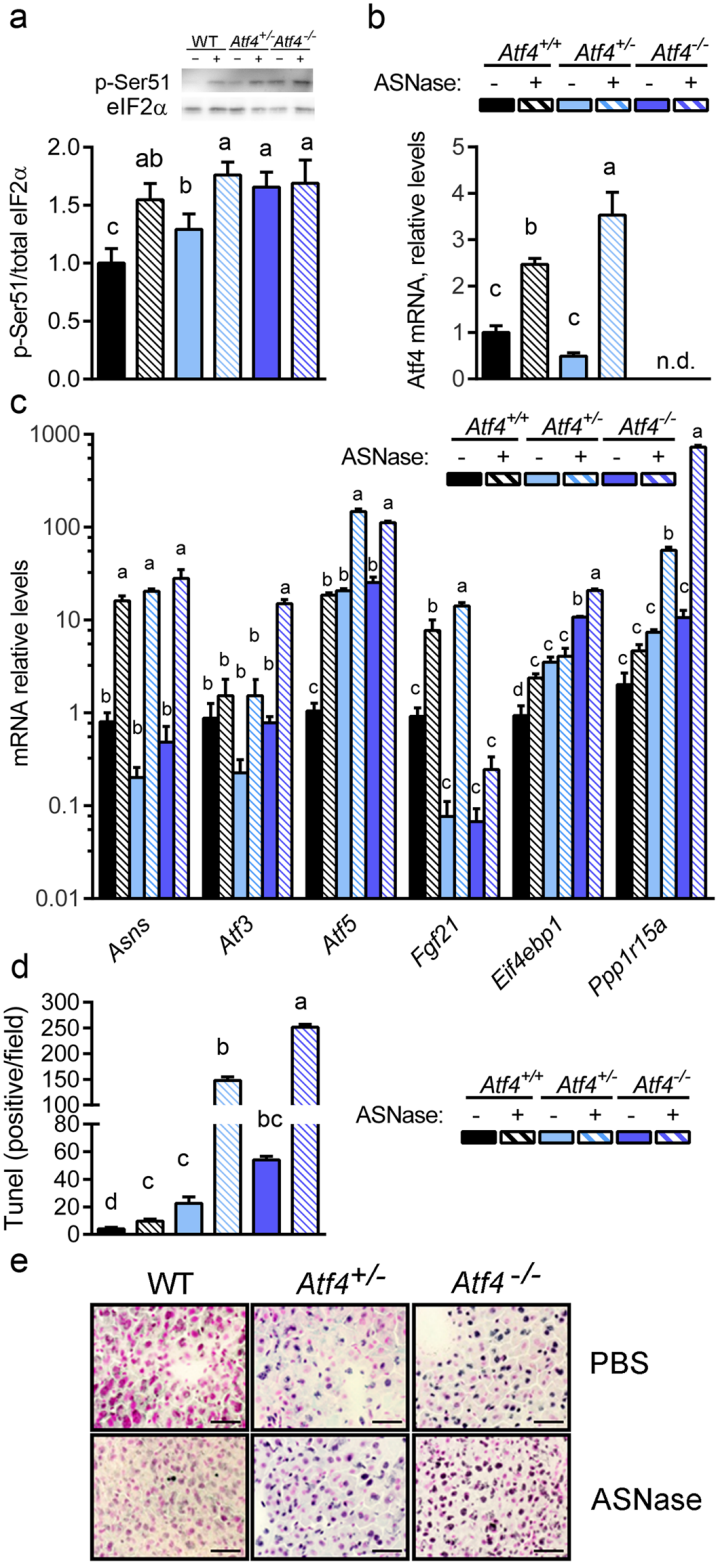
*Atf4* haploinsufficiency amplifies the hepatic AAR and liver damage in response to 8 daily injections of asparaginase (3 IU per gram body weight, ASNase). (**a**) Phospho-eIF2 alpha was measure by immunoblot analysis and quantified relative to total eIF2 alpha. (**b**) Gene expression of *Atf4* in liver as measured by RT-qPCR. n.d. = not detectible. (**c**) Hepatic expression of ISR genes *Asns*, *Atf3*, *Atf5*, *Fgf21*, *Eif4ebp1*, and *Ppp1r15a* in WT, *Atf4* ^+/−^ and *Atf4* ^−/−^ mice. (**d**) Apoptosis was ascertained in de-identified histological sections by manual counting of TUNEL-positive nuclei using Image J software. (**e**) Fragmented DNA visualization by TUNEL method in representative frozen liver sections (10 μm thick). Images taken at 40X magnification show visual features determined in WT, *Atf4* ^+/−^ and *Atf4* ^−/−^ mice. Scale bar represents 50 μm. Data are represented as the average value ± standard error of the mean, n = 3–6 per group. Means without a common letter are different according to Tukey post hoc analysis following ANOVA, P < 0.05. Full immunoblots are presented in Supplementary Fig. S7.

Hepatic p-eIF2 levels in PBS-injected *Atf4* ^+/−^ mice were elevated relative to *Atf4* ^+/+^ mice (Fig. 6a). Asparaginase further increased hepatic p-eIF2 in *Atf4* ^+/−^ mice above WT and equivalent to *Atf4* ^−/−^ mice. Consistent with this, relative mRNA levels of *Atf4* (Fig. 6b) and relative mRNA levels of ATF4 targets *Atf3*, *Atf5*, *Fgf21*, *Eif4ebp1*, and *Pppr1r15a (Gadd34)* in asparaginase-treated *Atf4* ^+/−^ mice were elevated above wild type mice (Fig. 6c). In contrast, livers from *Atf4* ^+/−^ mice treated with asparaginase expressed *Asns* similarly across strains. Consistent with the idea that loss of one *Atf4* allele reduced hepatoprotection, liver sections from *Atf4* ^+/−^ mice showed a high level of DNA fragmentation that was intermediate between *Atf4* ^+/+^ and *Atf4* ^−/−^ mice, indicating greater hepatic stress both basally and following asparaginase (Fig. 6d,e).

**Figure 7:**
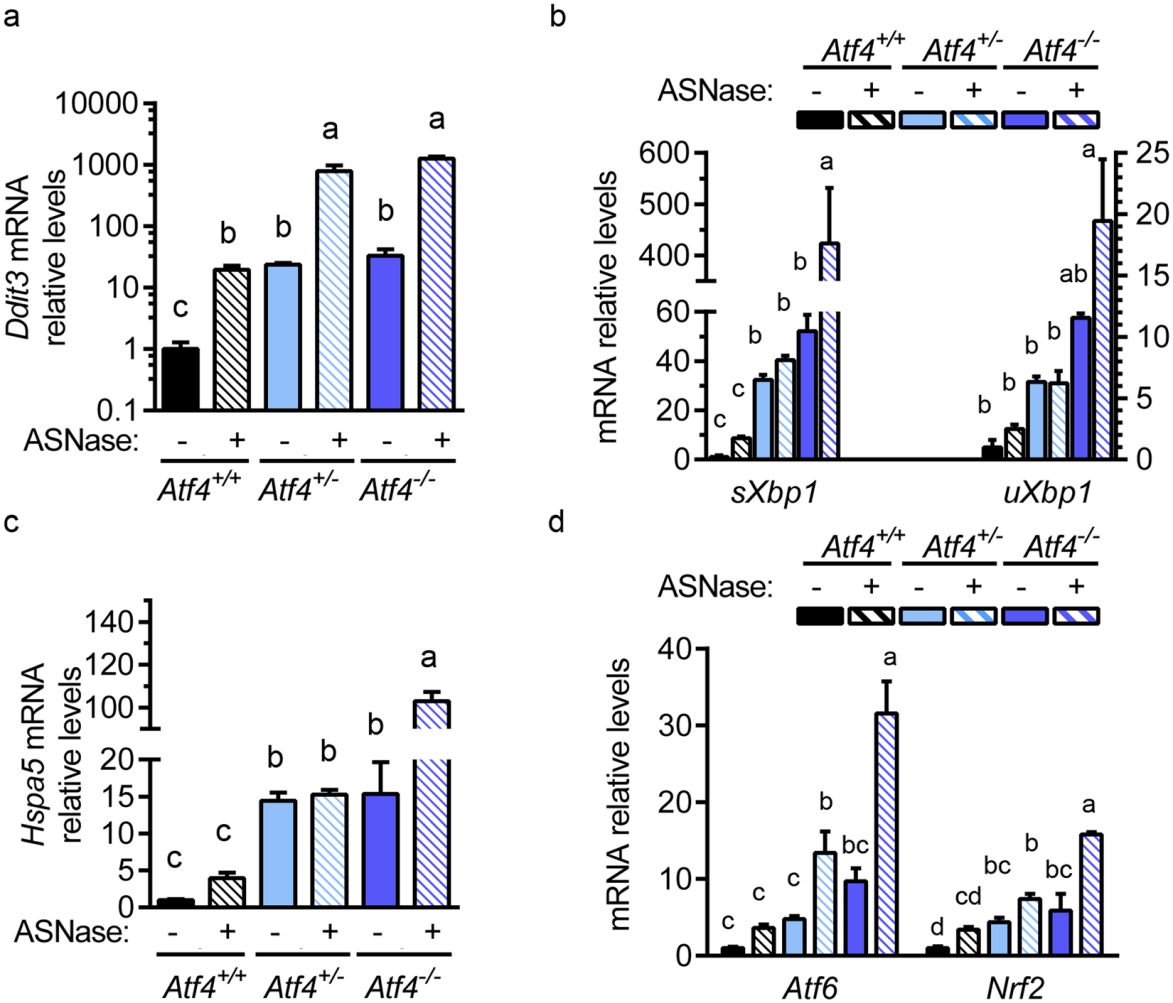
*Atf4* haploinsufficiency predisposes mice to amplified ER stress when treated with 8 daily injections of asparaginase (3 IU per gram body weight, ASNase). Gene expression levels of: (**a**) *Ddit3* (CHOP), (**b**) spliced *Xbp1* and unspliced *Xbp1*, (**c**) *Hspa5* (BiP/Grp78), and (**d**) *Atf6* and oxidative stress marker *Nrf2* were measured in liver by RT-qPCR. Data are represented as the average value ± standard error of the mean, n = 4–6 per group. Means without a common letter are different according to Tukey post hoc analysis following ANOVA, P < 0.05.

The overall amplified AAR induction in livers from *Atf4* ^+/−^ mice treated with asparaginase also corresponded with elevated ER stress. Supporting this idea, livers from both asparaginase-treated *Atf4* ^+/−^ and *Atf4* ^−/−^ mice showed increased *Ddit3* mRNA expression (Fig. 7a), and livers from asparaginase-treated *Atf4* ^−/−^ mice demonstrated increased *Xbp1* splicing (Fig. 7b), increased *Hspa5* (BiP/Grp78) mRNA expression (Fig. 7c) and increased *Atf6* mRNA expression as compared to PBS controls (Fig. 7d). Furthermore, gene expression of nuclear factor 2 (*Nrf2*), a marker of oxidative stress that is also identified as an ATF4 interacting protein^37^, was also elevated in asparaginase-treated *Atf4* ^+/−^ livers above asparaginase-treated *Atf4* ^+/+^ livers (Fig. 7d), suggesting that *Atf4* haploinsufficiency predisposes mice toward greater hepatic stress by asparaginase.

## Discussion

Liver dysfunction as a result of oxidative stress and hepatic steatosis is an asparaginase-associated adverse event that complicates remission induction and reduces treatment success^38–40^. A maladaptive GCN2/eIF2-directed ISR is proposed by our laboratory as a molecular basis for asparaginase-associated metabolic complications and hepatotoxicity^5, 6, 10–12^ but the role of ATF4 in this process is unknown. Herein we present a global view of how genetic loss of *Gcn2* versus *Atf4* influences hepatoprotection during asparaginase treatment by altering the liver transcriptome and signal transduction responses. First, we identify shared versus unique hepatic processes altered by GCN2 versus ATF4 status in the basal state and during chronic amino acid depletion by asparaginase. Second, we find that coordination of mTORC1 signaling by GCN2 does not require ATF4 or Sestrin2. Finally, we determine that both total and partial loss of *Atf4* expression is unable to self-limit the adaptive AAR to asparaginase, resulting in ER stress and greater cell death. These findings both confirm and challenge prevailing ideas on ATF4 function and add new mechanistic insight into how the liver adapts to amino acid deprivation.

This study provides the first view of the hepatic transcriptome on the background of global *Atf4* deletion and a first comparison to global deletion of *Gcn2*. This study strength must be viewed in balance with limitations that are inherent when studying mice with whole body gene deletion. Specifically, because ATF4 is a master regulator of metabolism and thermogenesis, global deletion of *Atf4* results in a lean phenotype with defects in eye, bone and blood cell development^27, 34–36^. Potential confounding influences of these health challenges on the liver transcriptome are acknowledged, particularly in light of the chronic treatment imposed. Indeed, these concerns formed the basis for pursuing experiments in *Atf4* ^+/−^ mice, which are healthy. In this regard, we note that the hepatic AAR response patterns following asparaginase exposure in *Atf4* ^+/−^ and *Atf4* ^−/−^ mice are directionally similar. Also, studies by our group and others using mice harboring a liver-specific deletion of *Atf4* show that its expression in liver plays important roles in regulating processes that were also identified in the current study such as cholesterol and lipid metabolism and oxidative stress responses^18, 41^. Furthermore, injection of asparaginase into obese mice shows that genetic ablation of *Atf4* in liver does not amplify mTORC1 signaling but instead amplifies a transcriptional signature similar to the current results^42^. Thus, the likelihood these outcomes are nonspecific or not relevant to ATF4 is low.

Mice lacking *Gcn2* do not show evidence of hepatic ER stress to asparaginase^11^. In contrast, *Atf4* ^−/−^ mice display an ER stress transcriptional signature in liver upon asparaginase treatment. These results suggest that ATF4 is necessary to self-limit the AAR in order to prevent inappropriate accumulation of an ER stress response transcriptional signature. Recently, we reported that in the absence of *Atf4*, ATF6 and CHOP assume auxiliary functions during pharmacological ER stress, driving the cell toward a maladaptive cell fate^18^. This report supports our current findings showing that in response to asparaginase treatment, expression of CHOP, *Atf6* and IRE1-directed splicing of *Xbp1* mRNA are potently induced in the livers of *Atf4*-deficient mice. These signals may allow other transcription factors to gain auxiliary function, directing the organism toward cellular death pathways rather than adaptive health outcomes. Additional studies are needed to more fully define how the binding partners versus targets of ATF4 direct cellular consequences to asparaginase. This is especially important because it will help explain why loss of one *Atf4* allele amplifies the ER stress transcriptional signature following asparaginase exposure.

Our lab was the first to identify hyperactivation of mTORC1 signaling in the *Gcn2* ^−/−^ condition during leucine deprivation and asparaginase^5, 31^, leading to the hypothesis that GCN2 or another ISR target coordinates amino acid sensing with mTORC1 signaling. One candidate is Sestrin2, an mTORC1 inhibitor regulated by ATF4 under conditions of amino acid deprivation or ER stress^23–25, 33^. Under conditions of amino acid deficiency, Sestrin2 becomes highly phosphorylated, promoting its interaction with a complex that subsequently blocks lysosomal localization of mTORC1^25, 43^. Using cells in culture, these studies identify a reciprocal relationship between the degree of Sesn2 phosphorylation and the degree of mTORC1 inhibition, specific to leucine availability^25^. However, others report that deprivation of other amino acids, such as glutamine, can also activate Sestrin2 inhibitory effect toward mTORC1^23^. Our results show Sestrin2 mRNA expression levels and phosphorylation of the protein are increased following asparaginase exposure even in the absence of *Gcn2*, *Atf4* or PERK activation and are not reciprocally related to mTORC1 signaling. We speculate that in the absence of GCN2-mediated signaling, Sestrin2 may be induced by a different profile of transcription factors such as CCAAT-enhancer-binding protein beta^44^ which in the current study was significantly elevated only in the livers of *Gcn2* ^−/−^ mice exposed to asparaginase (FPKM: *Gcn2* ^−/−^ PBS = 38; *Gcn2* ^−/−^ ASNase = 103, q = 0.0008). In the absence of *Atf4*, we suggest that auxiliary action by other ER stress-responsive transcription factors may promote Sestrin2 expression, based on experiments showing that both the PERK and the IRE1/XBP1 arms of the UPR contribute to regulating Sestrin2^45^. Experimental confirmation of these ideas still leaves open the question why is hepatic mTORC1 activity unleashed in *Gcn2* ^−/−^ but not *Atf4* ^−/−^ mice? Fafournoux’s group shows that GCN2-regulation of mTORC1 in cultured cells is ATF4-independent but involves phosphorylation of eIF2^46^. Our findings partially align with this idea, leaving open the need to resolve the contribution of eIF2 phosphorylation by other kinases to mTORC1 signaling versus independent control of mTORC1 by other transcription factors such as TRIB3^42^ versus regulation of mTORC1 secondary to the impact of *Gcn2* deletion on autophagy^12^.

In conclusion, this is the first comprehensive dataset identifying the role of *Gcn2* versus *Atf4* in control of hepatic transcriptome levels both basally and during chronic amino acid depletion. This information emphasizes how the function of the AAR contributes to the molecular basis of hepatoprotection in response to asparaginase. Application of these findings to dietary amino acid insufficiency in general is evident based on a recent study^47^ showing that multiple dietary restriction models elicit a gene expression signature sharing many of the same overrepresented canonical pathways as the current study and another report^48^ showing that removal of asparagine and/or glutamine from cells activates the ISR similar to dietary protein dilution. Future areas of investigation will use this information to develop personalized screening tools and/or discover targeted therapies to prevent adverse events to asparaginase or other therapies that utilize amino acid depletion as a treatment strategy.

## Methods

### Animals and Care

All animal protocols were reviewed and approved by the Institutional Animal Care and Use Committee at Rutgers, The State University of New Jersey and all animals received humane care according to the criteria outlined in the “Guide for the Care and use of Laboratory Animals” prepared by the National Academy of Sciences and published by the National Institute of Health (NIH publication 86–23 revised 1985) and ARRIVE (articulated at www.nc3rs.org.uk/ARRIVE). In these experiments, male and female C57BL/6J mice (8–12 wk old) carrying a homozygous deletion of *Gcn2* (*Gcn2* ^−/−^) or *Atf4 (Atf4* ^−/−^ *)* were used alongside wildtype controls (WT, *Atf4* ^+/+^) and *Atf4* heterozygote nulls (*Atf4* ^+/−^) (Jackson Laboratories, Bar Harbor, ME).

### RNA-Sequencing

RNA-Seq was performed at the JP Sulzberger Columbia Genome Center (Columbia University, NY, NY). Poly-A purified RNA was used with the TruSeq RNA Sample Prep Kit v2 from total RNA having a RIN ≥ 8.0. Libraries were sequenced on an Illumina HiSeq 2500 with a read depth of 30 million, 100 bp single-end reads. The resulting fastq files were subject to the Tuxedo bioinformatics pipeline^49, 50^. The reads were mapped to the mouse genome (mm10) with Tophat v2.0 then differences in gene expression were determined using Cuffdiff and evaluated according to drug treatment and genetic strain.

### Statistical Analysis

Global transcriptome analyses (n = 3 per group) were conducted in R using cummeRbund. Differentially expressed genes were considered to be statistically significant when the q value (or False Discovery Rate, FDR) was <0.1 (unadjusted p < 0.017). All other measurements utilized 3–6 animals per group and are reported as the average value ± standard error of the mean (SEM). Homoscedasticity was evaluated using Levene’s Test and data normality evaluated using Shapiro-Wilk-Test. Log transformation of data was performed when assumptions of distribution normality and homoscedasticity were violated. Differences between multiple treatment groups were analyzed by one and two factor ANOVA. When a statistically significant overall difference was detected, differences among individual means were evaluated using Tukey’s post-hoc test with an alpha level of P < 0.05 (STATISTICA; StatSoft, Tulsa, OK).

## Electronic supplementary material

Supplementary Information

Supplementary Table S2

Supplementary Table S3
