# Supplementary material for: Role of activating transcription factor 4 in the hepatic response to amino acid depletion by asparaginase
Source: Sci Rep. 2017 Apr 28;7:1272. doi: 10.1038/s41598-017-01041-7 (PMC5430736; doi:10.1038/s41598-017-01041-7)
Supplement: Supplementary file 1 — Supplementary Information [file 41598_2017_1041_MOESM1_ESM.pdf]

Supplemental Information

Role of activating transcription factor 4 in the hepatic response to amino acid depletion by asparaginase.

Rana J.T. Al-Baghdadi, Inna A. Nikonorova, Emily T. Mirek, Yongping Wang, Jinhee Park, William J. Belden, Ronald C. Wek, Tracy G. Anthony

Table of Contents:

1. Supporting Materials and Methods
2. Supporting References
3. Legend for Supplementary Figures
4. Supplementary Figure S1
5. Supplementary Figure S2
6. Supplementary Figure S3
7. Supplementary Figure S4
8. Supplementary Figure S5
9. Supplementary Figure S6
10. Supplementary Figure S7
11. Legend for Supplementary Tables
12. Supplementary Table S1
13. Supplementary Table S2 (separate Excel file)
14. Supplementary Table S3 (separate Excel file)

Supporting Materials and Methods

Animals. All male and female animals were on the C57BL/6J genetic background ($\geq$8 generations) and were bred and maintained at the Rutgers Bartlett animal care facility. *Gcn2*$^{-/-}$ mice were originally obtained from Douglas Cavener [1] whereas *Atf4*$^{-/-}$ mice (accession number: 013072 - *Atf4*^{tm1Tow}/J) were obtained from The Jackson Laboratory. Animals were genotyped by polymerase chain reaction (PCR) analysis of ear DNA using standard PCR methods. All mice were individually housed in clear plastic cages with corncob bedding in a temperature and humidity controlled room with a 12:12-h light-dark cycle. Mice were freely provided tap water and commercial rodent chow (5001 Laboratory Rodent Diet, LabDiet) at all times before and throughout experiments.

Experimental Design. Mice were administered daily intraperitoneal (i.p.) injections of native E. coli L-asparaginase (Elspar®, Deerfield, Illinois) in phosphate buffered saline (PBS) at 0 or 3.0 international units per gram body weight (IU/g BW) after the start of the light cycle as previously detailed [2]. Mice were euthanized by decapitation ~8h after the last injection.

Sample collection. Mice from all treatment groups were killed by decapitation ~8 h after the eighth daily injection. Trunk blood was collected to obtain serum. Tissues were rapidly dissected, rinsed in ice-cold PBS, blotted and weighed. One portion of each liver was snap-frozen in liquid nitrogen for further biochemical analyses. Frozen samples were stored at -80°C until analysis. A final portion was fixed in 4% paraformaldehyde as previously described [2].

Body composition. Body composition was determined by magnetic resonance using an EchoMRI instrument (Echo Medical Systems, Houston, TX).

RNA-Sequencing. Frozen liver samples were processed to obtain high quality RNA for RNA-Sequencing (RNA-Seq). Total RNA was extracted from frozen livers using NucleoSpin® RNA Kit (Macherey-Nagel, Newmann-Neander, Germany) followed by DNase treatment. The

A260/280 and 260/230 absorbance ratios were identified using NanoDrop1000 (Thermo Fisher Scientific, Wilmington, DE). RNA Integrity Number (RIN) was determined using an Agilent Bioanalyzer 2100 (Agilent Technologies, Waldbronn, Germany).

Data quality and differential gene expression analysis. The quality control analyses, Principle Component Analysis (PCA) and Volcano plot, were conducted using R (v3.2.2). Differences in gene expression were evaluated according to drug treatment and genetic strain. Fastq files were aligned to the mouse genome (mm10) using TopHat (v2.1.0) and Bowtie (v1.1.2) (http://ccb.jhu.edu/software.shtml). Mapped reads were submitted to Cufflinks (v2.2.1) using the default settings. The assembled transcript files were merged using Cuffmerg, quantified by Cuffdiff and then indexed and visualized using CummeRbund (v2.12.0). Average FPKM values (n=3) were used to calculate differences in expression between different strains and treatment groups.

To analyze basal differences between WT (*Atf4*$^{+/+}$ *and Gcn2*$^{+/+}$), *Gcn2*$^{-/-}$ and *Atf4*$^{-/-}$ strains and their response to ASNase treatment we considered only genes that had FPKM values of more than 3 in either of the strain/treatment group and with absolute values of $\log_2$(fold change between compared groups) of more than 1.5. The resulting lists of genes were sorted to categories by Venn method (Fig 2A,C). The list of genes in each category was then analyzed using Ingenuity Pathway Analysis software (Qiagen, Redwood City, CA) to identify pathways that might be affected by the loss of either *Gcn2* or *Atf4*. Gene lists that were placed into shared categories (B, F, H, I, J) were subjected to cluster analysis using CIMminer (https://www.discover.nci.nih.gov/cimminer/) according to their log2(fold change) values to visualize directionality of the change in gene expression and the extent to which it changes.

Integrated Genome Viewer (IGV) (v2.3) was used to confirm the deletion of the *Gcn2* and *Atf4* genes (https://www.broadinstitute.org/igv/v1.2).

Hepatic mRNA expression levels by Reverse Transcriptase Quantitative PCR. Total RNA was extracted as described above. 1µg of purified RNA was reverse transcribed using the High-Capacity cDNA Reverse Transcription Kit (Applied Biosystems, Foster City, CA). Relative mRNA expression levels were determined by quantitative PCR using TaqMan reagents and the StepOnePlus Real-Time PCR System (Applied Biosystems, Foster, CA). Each mRNA from a single biological sample was measured in triplicate and normalized to 18S ribosomal RNA. Results were obtained by the comparative Ct method and are expressed as fold change with respect to the experimental control as previously detailed [3]. . All primer/probe assays were ordered from Life Technologies except for spliced Xbp1 in which the primers (Forward: 5′-GAGTCCGCAGCAGGTG-3′; Reverse: 5′-CTCTGGGAGTTCCTCCAGACT-3′) were ordered from Integrated DNA Technologies and used with Universal Probe Library, probe #60 from Roche Diagnostics.

Immunoblot analysis. Tissue lysates were prepared as previously described [2,4]. The following primary antibodies were purchased from Cell Signaling Technology (Beverly, MA): phospho-eIF2α (#3597); phospho-Akt Thr308 (#9275); total AKT (#9272); phospho-P70S6K Thr389 (#9205); total S6K1 (#9202); phospho-PERK Thr980 (#3179); total PERK (#3192); and GAPDH (#2118). Other primary antibodies included: total eIF2α (#sc11386, Santa Cruz Biotechnology, Dallas, TX); CHOP (#sc7351 Santa Cruz Biotechnology, Dallas, TX); 4EBP1, (#A300-501A, Bethyl Laboratories, Montgomery, TX); SESN2 (#10082-224, ProteinTech, Rosemont, IL). Immunoblot membranes were processed and developed using enhanced chemiluminesence kit (Amersham Biosciences, Pittsburgh, PA). Chemiluminescence signal intensities were digitally captured using a FluorChem M multiplex imager (Protein Simple) and band densities were quantitated using Carestream Molecular Imaging Software (version 5.0).

Histology. A portion of each fixed liver sample was paraffin-embedded and 5 µM thick sections were mounted on microscope slides. Slides were stained with hematoxylin, counterstained with

eosin, dehydrated and mounted for light microscopy observation and also evaluated for DNA fragmentation by TUNEL assay as described [5]. Frozen sections (10 μM thick) from fixed liver were stained with Oil Red O to visualize neutral lipid content. Liver triglycerides were also measured biochemically using a commercially available kit (Biovision, Milpitas, CA).

Image acquisition and processing. Bar graphs were created using GraphPad Prism 6 software and exported tagged image files (TIFF) with a 300 dpi resolution and RGB color model. Venn diagrams and tables were created using Microsoft Office software. Digitally captured histology and immunoblots were exported as 8 bit TIFF. Data were assembled into multi-panel figure displays using Adobe Photoshop.

Supplementary Methods References

1 Zhang, P. *et al.* The GCN2 eIF2alpha kinase is required for adaptation to amino acid deprivation in mice. *Mol Cell Biol* **22**, 6681-6688 (2002).

2 Wilson, G. J. *et al.* GCN2 is required to increase fibroblast growth factor 21 and maintain hepatic triglyceride homeostasis during asparaginase treatment. *Am J Physiol Endocrinol Metab*, ajpendo 00361 02014, doi:10.1152/ajpendo.00361.2014 (2015).

3 Wilson, G. J., Bunpo, P., Cundiff, J. K., Wek, R. C. & Anthony, T. G. The eukaryotic initiation factor 2 kinase GCN2 protects against hepatotoxicity during asparaginase treatment. *Am J Physiol Endocrinol Metab* **305**, E1124-1133, doi:10.1152/ajpendo.00080.2013 (2013).

4 Bunpo, P. *et al.* GCN2 protein kinase is required to activate amino acid deprivation responses in mice treated with the anti-cancer agent L-asparaginase. *J Biol Chem* **284**, 32742-32749, doi:10.1074/jbc.M109.047910 (2009).

5 Bunpo, P. *et al.* The eIF2 kinase GCN2 is essential for the murine immune system to adapt to amino acid deprivation by asparaginase. *J Nutr* **140**, 2020-2027, doi:10.3945/jn.110.129197 (2010).

Legend for Supplementary Figures

Supplementary Figure S1: Body weight and body composition before and after 8 daily injections of asparaginase (3 IU per gram body weight, ASNase) or phosphate buffered saline excipient (PBS) in wild type mice (WT) or mice deleted for *Gcn2* (*Gcn2*$^{-/-}$) or *Atf4* (*Atf4*$^{-/-}$). (a) Percent of body fat mass and percent of body lean mass was measured by EchoMRI before the treatment commenced (Start) and after final injections (End). (b) Percent body weight change between Start and End in WT, *Gcn2*$^{-/-}$ and *Atf4*$^{-/-}$ mice treated with 8 daily intraperitoneal injections of ASNase or PBS. Data are represented as the average value ± standard error of the mean, n=4-6 per group. Means without a common letter are different according to Tukey post hoc analysis following ANOVA, P<0.05.

Supplementary Figure S2: Deletion of *Gcn2* and *Atf4* genes in mice. (a) RNA read density plots using the Integrated Genome Viewer from the Broad Institute confirm the absence of *Gcn2* exon 12 in *Gcn2*$^{-/-}$ mice. (b) RNA read density plots confirm the absence of *Atf4* in *Atf4*$^{-/-}$ mice. (c) *Atf4* expression was also assessed by RT-qPCR. Data are represented as the average value ± standard error of the mean, n=3 per group. Means without a common letter are different according to Tukey post hoc analysis following ANOVA, P<0.05.

Supplementary Figure S3: Global changes in hepatic gene expression after 8 daily injections of asparaginase (3 IU per gram body weight, ASNase) or phosphate buffered saline excipient (PBS) in wild type mice (WT, *Atf4*$^{+/+}$ *and Gcn2*$^{+/+}$) or mice deleted for *Gcn2* (*Gcn2*$^{-/-}$) or *Atf4* (*Atf4*$^{-/-}$). (a) Cross-comparison Volcano plots show the proportion of genes that differ significantly (highlighted in red) among PBS and ASNase treatment conditions. X-axis represents Log2 (fold change) and Y axis represents the –log(P-value). (b) Principle Component

Analysis (PCA) plot of transcriptome changes induced by ASNase. Data represent the average value of n=3 per group.

Supplementary Figure S4: Cluster diagrams of differentially expressed genes from shared Venn categories (B,F,H, I. J) were generated using CIMminer online tool.

Supplementary Figure S5: Relative levels of *Eif4ebp1* (a) and *Sestrin2* (b) mRNA measured in liver after 8 daily injections of asparaginase (3 IU per gram body weight, ASNase) or phosphate buffered saline excipient (PBS) in wild type mice (WT, *Atf4*$^{+/+}$ *and Gcn2*$^{+/+}$) or mice deleted for *Gcn2* (*Gcn2*$^{-/-}$) or *Atf4* (*Atf4*$^{-/-}$). Data are represented as the average value ± standard error of the mean, n=3 per group. Means without a common letter are different according to Tukey post hoc analysis following ANOVA, P<0.05.

Supplementary Figure S6: Body composition and tissue responses to asparaginase. (a) Percent body weight change in WT, *Atf4*$^{+/-}$ and *Atf4*$^{-/-}$ mice treated with 8 daily intraperitoneal injections of asparaginase (3 IU per gram body weight, ASNase) or equivolume phosphate buffered saline (PBS) excipient and killed 8 h after the final injection. (b) Percentage of body fat and lean mass before (Start) and after (End) treatment measured using EchoMRI. **, main effect of strain; i.e., *Atf4*$^{-/-}$ mice were leaner than other mouse strains at Start; #, drug x strain interaction; i.e., ASNase reduced fat mass in *Atf4*$^{-/-}$ mice only. (c) Percent weight change of liver, pancreas, and spleen relative to body weight. +, main effect of drug; ASNase reduced spleen weight in all strains. (d) Lipid accumulation in livers of mice. Oil Red O stained liver sections (5μm thick) show neutral lipid accumulation. Images were taken at 40X magnification

and the scale bar represents 50 μm. (e) Triglyceride concentrations in the livers of mice measured biochemically. Data are represented as the average value ± standard error of the mean, n=4-6 per group. Means without a common letter are different according to Tukey post hoc analysis following ANOVA, $P<0.05$.

Supplementary Figure 7. Full immunoblots for all representative images displayed in Figures 3-6.

Supplementary Figure S1

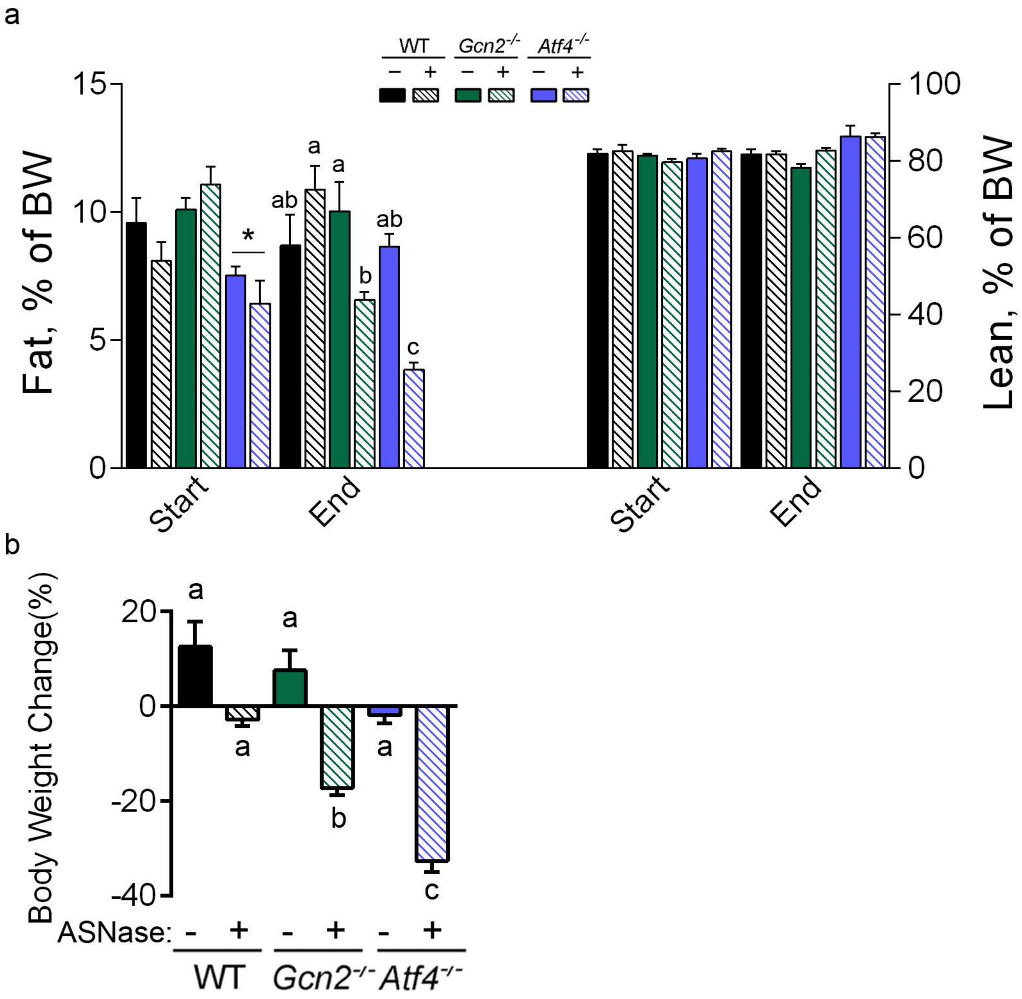

a

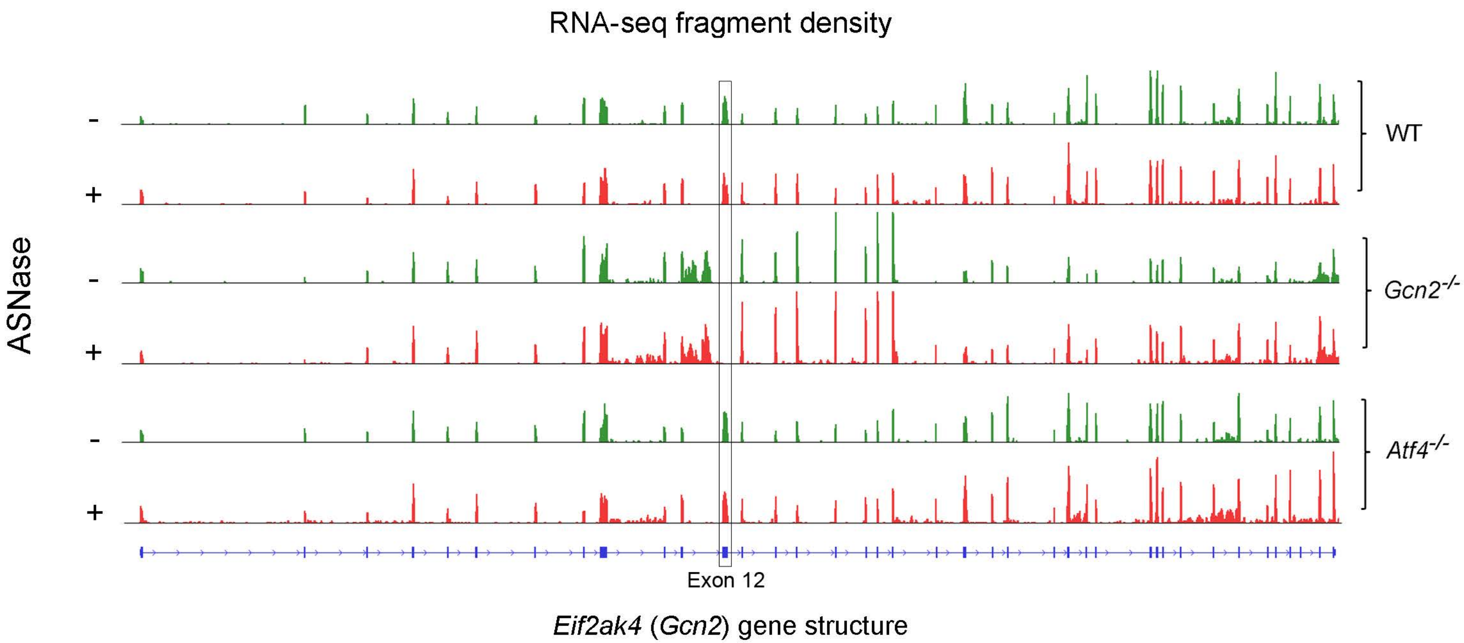
RNA-seq fragment density
ASNase
-
+
-
+
-
+
WT
Gcn2-/-
Atf4-/-
Exon 12
Eif2ak4 (Gcn2) gene structure

b

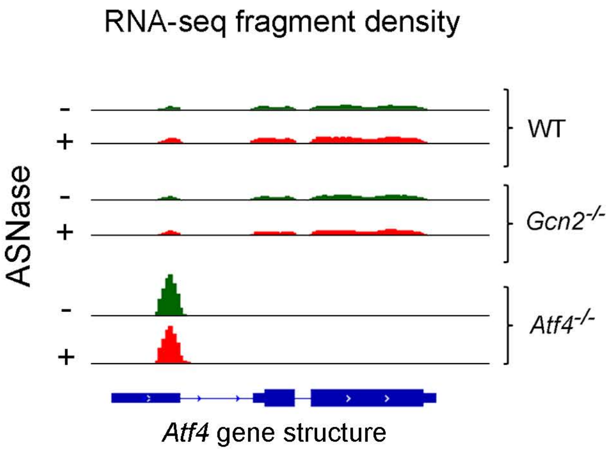
RNA-seq fragment density
ASNase
-
+
-
+
-
+
WT
Gcn2-/-
Atf4-/-
Atf4 gene structure

c

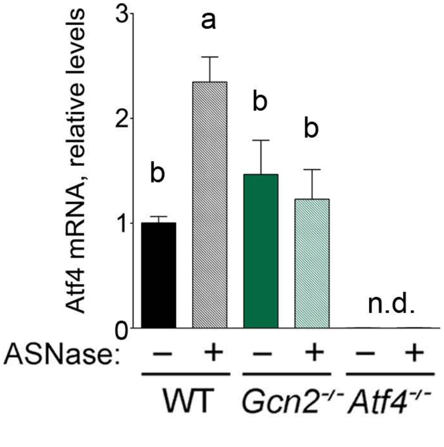
Atf4 mRNA, relative levels
0
1
2
3
b
a
b
b
n.d.
ASNase:
–
+
–
+
–
+
WT
Gcn2-/-
Atf4-/-

Supplementary Figure S3

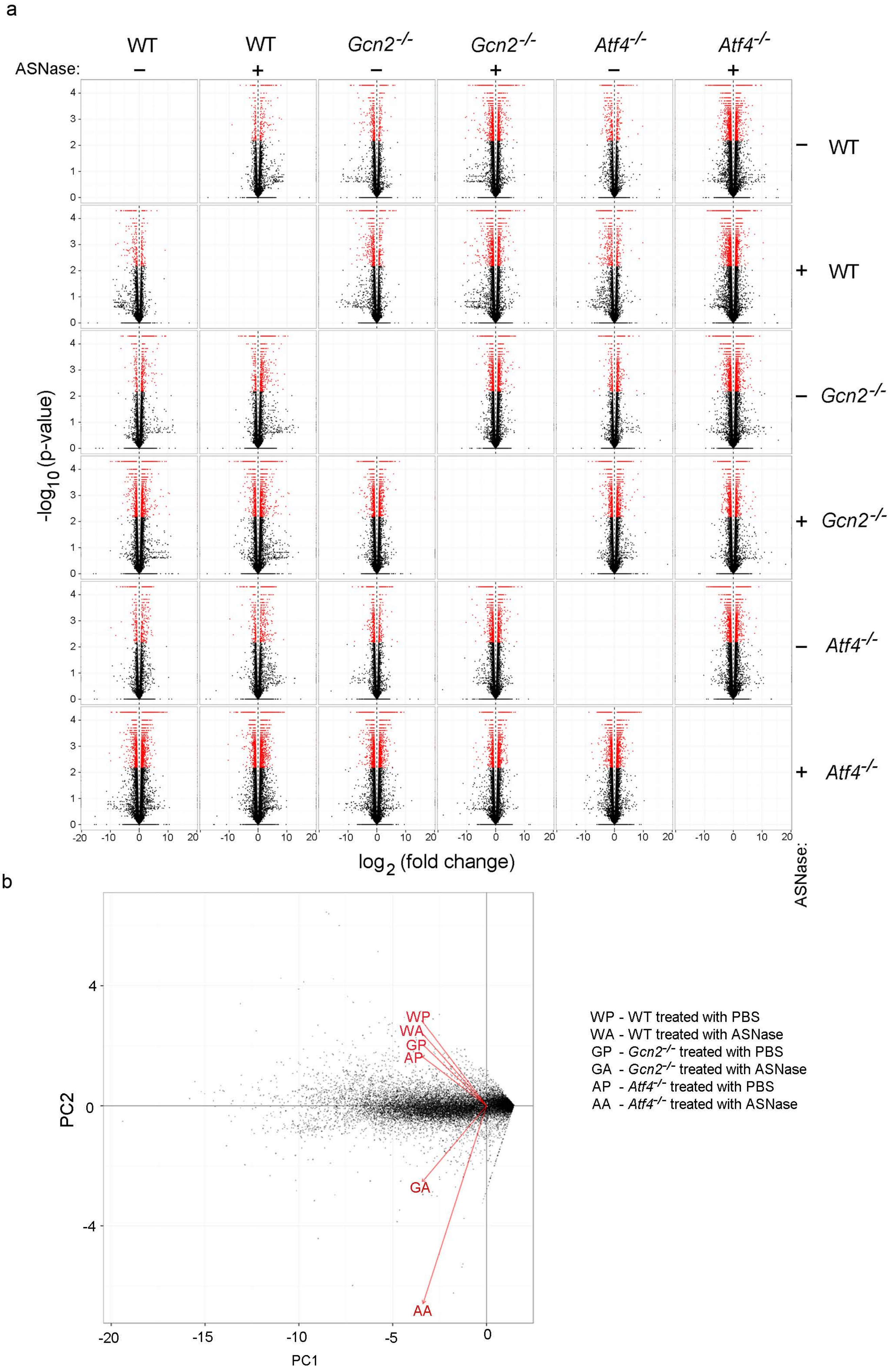

Venn Category B

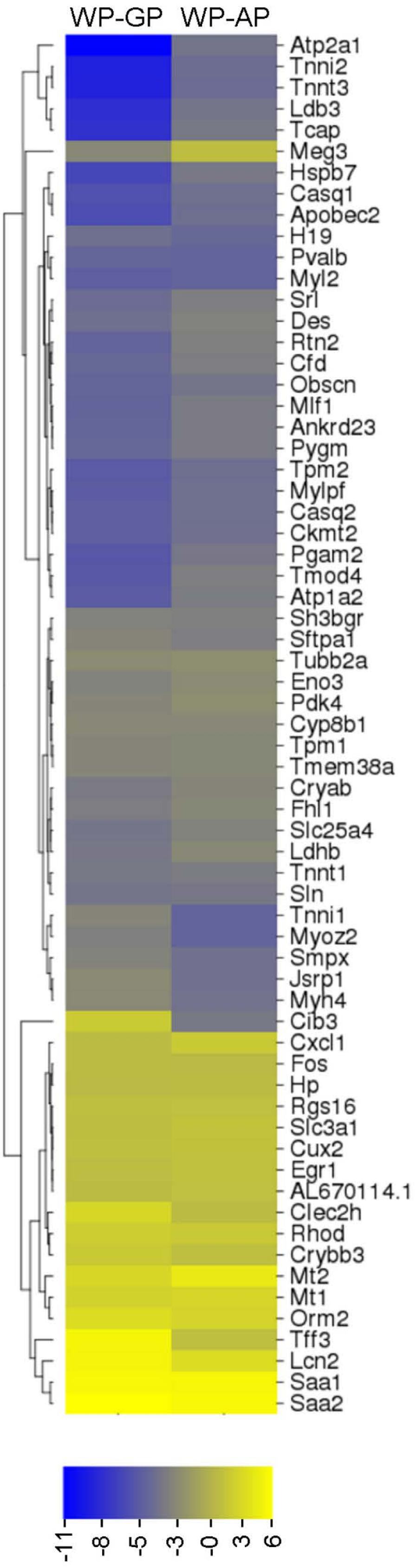

Legend:

WP = Wild type PBS

WA = Wild type ASNase

GP = *Gcn2-/-* PBS

GA = *Gcn2-/-* ASNase

AP = *Atf4-/-* PBS

AA = *Atf4-/-* ASNase

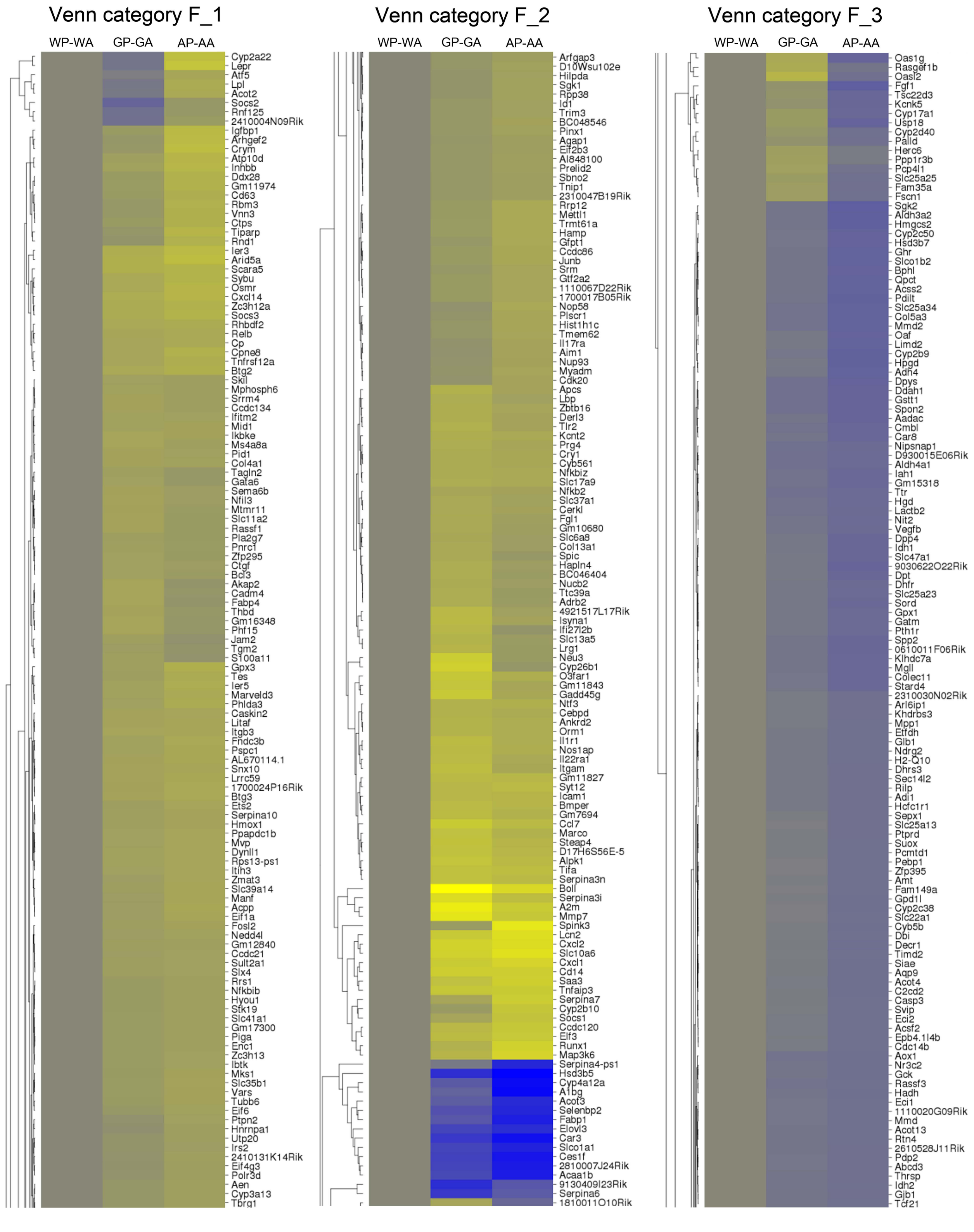

Venn category F_1
WP-WA
GP-GA
AP-AA
Cyp2a22
Lepr
Atf5
Lpl
Acot2
Socs2
Rnf125
2410004N09Rik
Igfbp1
Arhgef2
Crym
Atp10d
Inhbb
Ddx28
Gm11974
Cd63
Rbm3
Vnn3
Ctps
Tiparp
Rnd1
Ier3
Arid5a
Scara5
Sybu
Osmr
Cxcl14
Zc3h12a
Socs3
Rhbdf2
Relb
Cp
Cpne8
Tnfrsf12a
Btg2
Skil
Mphosph6
Srrm4
Ccdc134
Ifitm2
Mid1
Ikbke
Ms4a8a
Pid1
Col4a1
Tagln2
Gata6
Sema6b
Nfil3
Mtmr11
Slc11a2
Rassf1
Pla2g7
Pnrc1
Zfp295
Ctgf
Bcl3
Akap2
Cadm4
Fabp4
Thbd
Gm16348
Phf15
Jam2
Tgm2
S100a11
Gpx3
Tes
Ier5
Marveld3
Phlda3
Caskin2
Litaf
Itgb3
Fndc3b
Pspc1
AL670114.1
Snx10
Lrrc59
1700024P16Rik
Btg3
Ets2
Serpina10
Hmox1
Ppapdc1b
Mvp
Dynll1
Rps13-ps1
Itih3
Zmat3
Slc39a14
Manf
Acpp
Eif1a
Fosl2
Nedd4l
Gm12840
Ccdc21
Sult2a1
Slx4
Rrs1
Nfkbib
Hyou1
Stk19
Slc41a1
Gm17300
Piga
Enc1
Zc3h13
Ibtk
Mks1
Slc35b1
Vars
Tubb6
Eif6
Ptpn2
Hnrnpa1
Utp20
Irs2
2410131K14Rik
Eif4g3
Polr3d
Aen
Cyp3a13
Tbrg1
Venn category F_2
WP-WA
GP-GA
AP-AA
Arfgap3
D10Wsu102e
Hilpda
Sgk1
Rpp38
Id1
Trim3
BC048546
Pinx1
Agap1
Eif2b3
AI848100
Prelid2
Sbno2
Tnip1
2310047B19Rik
Rrp12
Mettl1
Trmt61a
Hamp
Gfpt1
Ccdc86
Junb
Srm
Gtf2a2
1110067D22Rik
1700017B05Rik
Nop58
Plscr1
Hist1h1c
Tmem62
Il17ra
Aim1
Nup93
Myadm
Cdk20
Apcs
Lbp
Zbtb16
Derl3
Tlr2
Kcnt2
Prg4
Cry1
Cyb561
Nfkbiz
Slc17a9
Nfkb2
Slc37a1
Cerkl
Fgl1
Gm10680
Slc6a8
Col13a1
Spic
Hapln4
BC046404
Nucb2
Ttc39a
Adrb2
4921517L17Rik
Isyna1
Ifi27l2b
Slc13a5
Lrg1
Neu3
Cyp26b1
O3far1
Gm11843
Gadd45g
Ntf3
Cebpd
Ankrd2
Orm1
Il1r1
Nos1ap
Il22ra1
Itgam
Gm11827
Syt12
Icam1
Bmper
Gm7694
Ccl7
Marco
Steap4
D17H6S56E-5
Alpk1
Tifa
Serpina3n
Boll
Serpina3i
A2m
Mmp7
Spink3
Lcn2
Cxcl2
Slc10a6
Cxcl1
Cd14
Saa3
Tnfaip3
Serpina7
Cyp2b10
Socs1
Ccdc120
Elf3
Runx1
Map3k6
Serpina4-ps1
Hsd3b5
Cyp4a12a
A1bg
Acot3
Selenbp2
Fabp1
Elovl3
Car3
Slco1a1
Ces1f
2810007J24Rik
Acaa1b
9130409I23Rik
Serpina6
1810011O10Rik
Venn category F_3
WP-WA
GP-GA
AP-AA
Oas1g
Rasgef1b
Oasl2
Fgf1
Tsc22d3
Kcnk5
Cyp17a1
Usp18
Cyp2d40
Palld
Herc6
Ppp1r3b
Pcp4l1
Slc25a25
Fam35a
Fscn1
Sgk2
Aldh3a2
Hmgcs2
Cyp2c50
Hsd3b7
Ghr
Slco1b2
Bphl
Qpct
Acss2
Pdilt
Slc25a34
Col5a3
Mmd2
Oaf
Limd2
Cyp2b9
Hpgd
Adh4
Dpys
Ddah1
Gstt1
Spon2
Aadac
Cmbl
Car8
Nipsnap1
D930015E06Rik
Aldh4a1
Iah1
Gm15318
Ttr
Hgd
Lactb2
Nit2
Vegfb
Dpp4
Idh1
Slc47a1
9030622O22Rik
Dpt
Dhfr
Slc25a23
Sord
Gpx1
Gatm
Pth1r
Spp2
0610011F06Rik
Klhdc7a
Mgll
Colec11
Stard4
2310030N02Rik
Arl6ip1
Khdrbs3
Mpp1
Etfdh
Glb1
Ndrg2
H2-Q10
Dhrs3
Sec14l2
Rilp
Adi1
Hcfc1r1
Sepx1
Slc25a13
Ptprd
Suox
Pcmtd1
Pebp1
Zfp395
Amt
Fam149a
Gpd1l
Cyp2c38
Slc22a1
Cyb5b
Dbi
Decr1
Timd2
Siae
Aqp9
Acot4
C2cd2
Casp3
Svip
Eci2
Acsf2
Epb4.1l4b
Cdc14b
Aox1
Nr3c2
Gck
Rassf3
Hadh
Eci1
1110020G09Rik
Mmd
Acot13
Rtn4
2610528J11Rik
Pdp2
Abcd3
Thrsp
Idh2
Gjb1
Tcf21

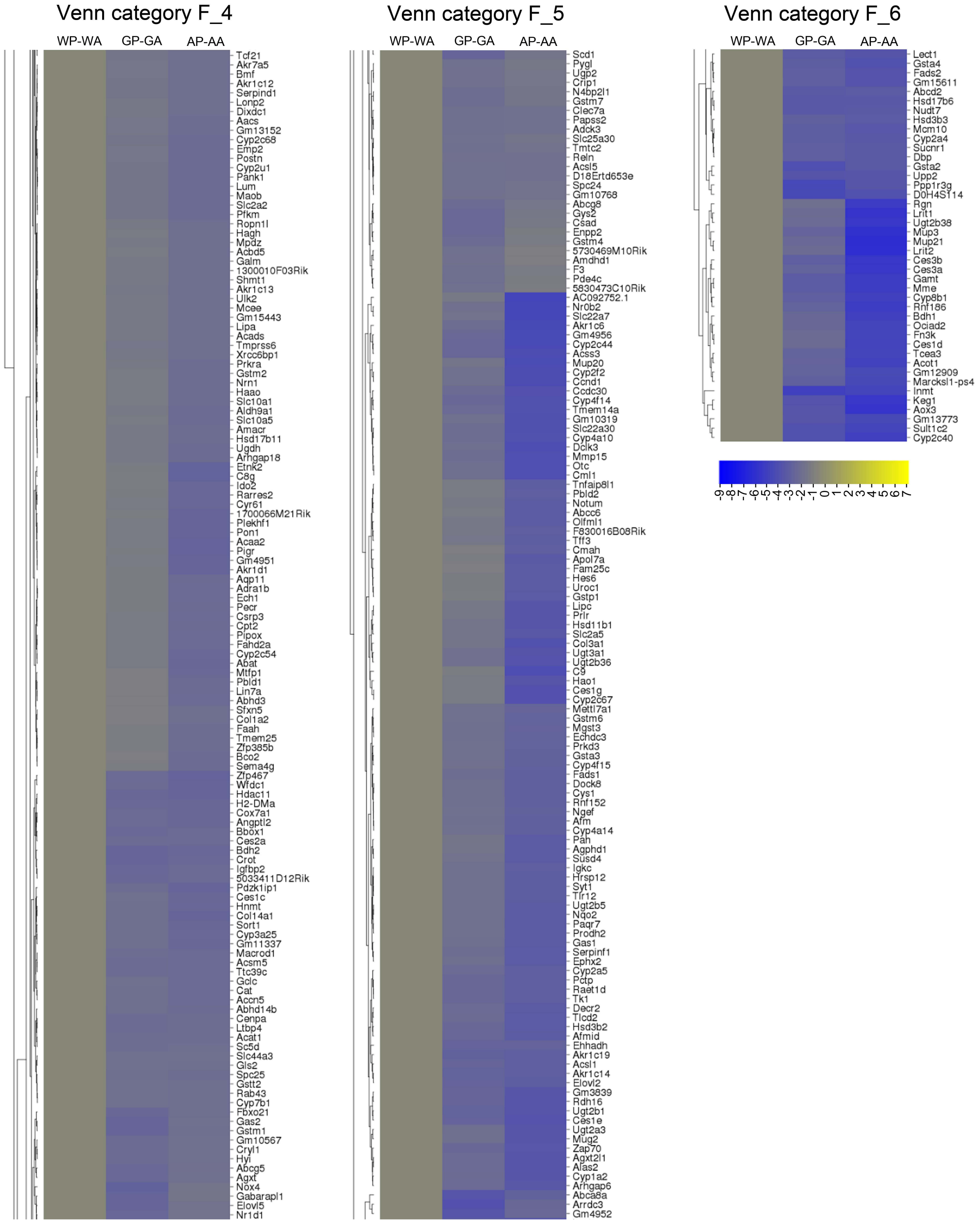
Venn category F_4
WP-WA
GP-GA
AP-AA
Tcf21
Akr7a5
Bmf
Akr1c12
Serpind1
Lonp2
Dixdc1
Aacs
Gm13152
Cyp2c68
Emp2
Postn
Cyp2u1
Pank1
Lum
Maob
Slc2a2
Pfkm
Ropn1l
Hagh
Mpdz
Acbd5
Galm
1300010F03Rik
Shmt1
Akr1c13
Ulk2
Mcee
Gm15443
Lipa
Acads
Tmprss6
Xrcc6bp1
Prkra
Gstm2
Nrn1
Haao
Slc10a1
Aldh9a1
Slc10a5
Amacr
Hsd17b11
Ugdh
Arhgap18
Etnk2
C8g
Ido2
Rarres2
Cyr61
1700066M21Rik
Plekhf1
Pon1
Acaa2
Pigr
Gm4951
Akr1d1
Aqp11
Adra1b
Ech1
Pecr
Csrp3
Cpt2
Pipox
Fahd2a
Cyp2c54
Abat
Mtfp1
Pbld1
Lin7a
Abhd3
Sfxn5
Col1a2
Faah
Tmem25
Zfp385b
Bco2
Sema4g
Zfp467
Wfdc1
Hdac11
H2-DMa
Cox7a1
Angptl2
Bbox1
Ces2a
Bdh2
Crot
Igfbp2
5033411D12Rik
Pdzk1ip1
Ces1c
Hnmt
Col14a1
Sort1
Cyp3a25
Gm11337
Macrod1
Acsm5
Ttc39c
Gclc
Cat
Accn5
Abhd14b
Cenpa
Ltbp4
Acat1
Sc5d
Slc44a3
Gls2
Spc25
Gstt2
Rab43
Cyp7b1
Fbxo21
Gas2
Gstm1
Gm10567
Cryl1
Hyi
Abcg5
Agxt
Nox4
Gabarapl1
Elovl5
Nr1d1
Venn category F_5
WP-WA
GP-GA
AP-AA
Scd1
Pygl
Ugp2
Crip1
N4bp2l1
Gstm7
Clec7a
Papss2
Adck3
Slc25a30
Tmtc2
Reln
Acsl5
D18Ertd653e
Spc24
Gm10768
Abcg8
Gys2
Csad
Enpp2
Gstm4
5730469M10Rik
Amdhd1
F3
Pde4c
5830473C10Rik
AC092752.1
Nr0b2
Slc22a7
Akr1c6
Gm4956
Cyp2c44
Acss3
Mup20
Cyp2f2
Ccnd1
Ccdc30
Cyp4f14
Tmem14a
Gm10319
Slc22a30
Cyp4a10
Dclk3
Mmp15
Otc
Cml1
Tnfaip8l1
Pbld2
Notum
Abcc6
Olfml1
F830016B08Rik
Tff3
Cmah
Apol7a
Fam25c
Hes6
Uroc1
Gstp1
Lipc
Prlr
Hsd11b1
Slc2a5
Col3a1
Ugt3a1
Ugt2b36
C9
Hao1
Ces1g
Cyp2c67
Mettl7a1
Gstm6
Mgst3
Echdc3
Prkd3
Gsta3
Cyp4f15
Fads1
Dock8
Cys1
Rnf152
Ngef
Afm
Cyp4a14
Pah
Agphd1
Susd4
Igkc
Hrsp12
Syt1
Tlr12
Ugt2b5
Nqo2
Paqr7
Prodh2
Gas1
Serpinf1
Ephx2
Cyp2a5
Pctp
Raet1d
Tk1
Decr2
Tlcd2
Hsd3b2
Afmid
Ehhadh
Akr1c19
Acsl1
Akr1c14
Elovl2
Gm3839
Rdh16
Ugt2b1
Ces1e
Ugt2a3
Mug2
Zap70
Agxt2l1
Alas2
Cyp1a2
Arhgap6
Abca8a
Arrdc3
Gm4952
Venn category F_6
WP-WA
GP-GA
AP-AA
Lect1
Gsta4
Fads2
Gm15611
Abcd2
Hsd17b6
Nudt7
Hsd3b3
Mcm10
Cyp2a4
Sucnr1
Dbp
Gsta2
Upp2
Ppp1r3g
D0H4S114
Rgn
Lrit1
Ugt2b38
Mup3
Mup21
Lrit2
Ces3b
Ces3a
Gamt
Mme
Cyp8b1
Rnf186
Bdh1
Ociad2
Fn3k
Ces1d
Tcea3
Acot1
Gm12909
Marcksl1-ps4
Inmt
Keg1
Aox3
Gm13773
Sult1c2
Cyp2c40
-9 -8 -7 -6 -5 -4 -3 -2 -1 0 1 2 3 4 5 6 7

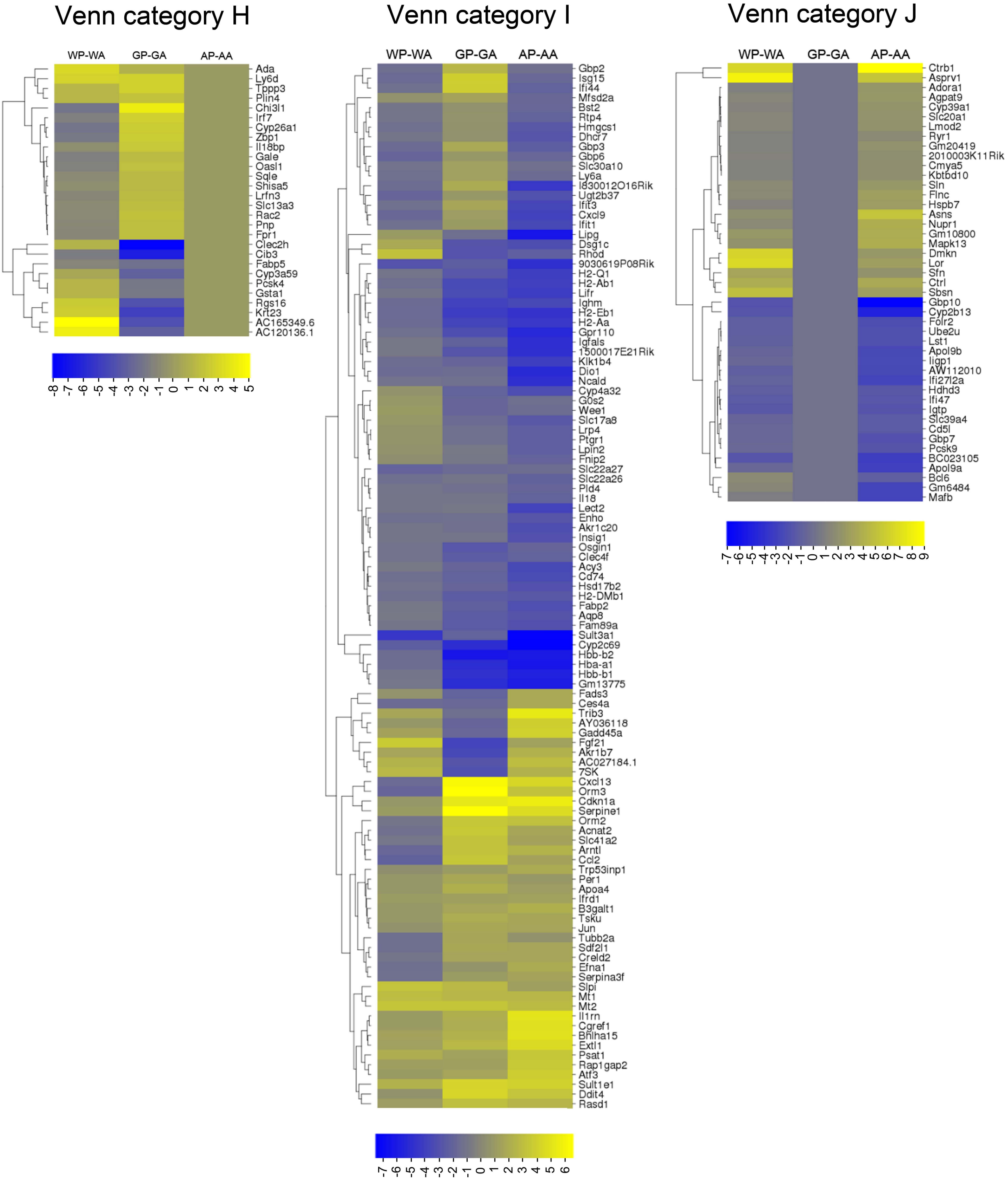

Venn category H
WP-WA
GP-GA
AP-AA
Ada
Ly6d
Tppp3
Plin4
Chi3l1
Irf7
Cyp26a1
Zbp1
Il18bp
Gale
Oasl1
Sqle
Shisa5
Lrfn3
Slc13a3
Rac2
Pnp
Fpr1
Clec2h
Cib3
Fabp5
Cyp3a59
Pcsk4
Gsta1
Rgs16
Krt23
AC165349.6
AC120136.1
-8 -7 -6 -5 -4 -3 -2 -1 0 1 2 3 4 5
Venn category I
WP-WA
GP-GA
AP-AA
Gbp2
Isg15
Ifi44
Mfsd2a
Bst2
Rtp4
Hmgcs1
Dhcr7
Gbp3
Gbp6
Slc30a10
Ly6a
I830012O16Rik
Ugt2b37
Ifit3
Cxcl9
Ifit1
Lipg
Dsg1c
Rhod
9030619P08Rik
H2-Q1
H2-Ab1
Lifr
Ighm
H2-Eb1
H2-Aa
Gpr110
Igfals
1500017E21Rik
Klk1b4
Dio1
Ncald
Cyp4a32
G0s2
Wee1
Slc17a8
Lrp4
Ptgr1
Lpin2
Fnip2
Slc22a27
Slc22a26
Pld4
Il18
Lect2
Enho
Akr1c20
Insig1
Osgin1
Clec4f
Acy3
Cd74
Hsd17b2
H2-DMb1
Fabp2
Aqp8
Fam89a
Sult3a1
Cyp2c69
Hbb-b2
Hba-a1
Hbb-b1
Gm13775
Fads3
Ces4a
Trib3
AY036118
Gadd45a
Fgf21
Akr1b7
AC027184.1
7SK
Cxcl13
Orm3
Cdkn1a
Serpine1
Orm2
Acnat2
Slc41a2
Arntl
Ccl2
Trp53inp1
Per1
Apoa4
Ifrd1
B3galt1
Tsku
Jun
Tubb2a
Sdf2l1
Creld2
Efna1
Serpina3f
Slpi
Mt1
Mt2
Il1rn
Cgref1
Bhlha15
Extl1
Psat1
Rap1gap2
Atf3
Sult1e1
Ddit4
Rasd1
-7 -6 -5 -4 -3 -2 -1 0 1 2 3 4 5 6
Venn category J
WP-WA
GP-GA
AP-AA
Ctrb1
Asprv1
Adora1
Agpat9
Cyp39a1
Slc20a1
Lmod2
Ryr1
Gm20419
2010003K11Rik
Cmya5
Kbtbd10
Sln
Flnc
Hspb7
Asns
Nupr1
Gm10800
Mapk13
Dmkn
Lor
Sfn
Ctrl
Sbsn
Gbp10
Cyp2b13
Folr2
Ube2u
Lst1
Apol9b
Iigp1
AW112010
Ifi27l2a
Hdhd3
Ifi47
Igtp
Slc39a4
Cd5l
Gbp7
Pcsk9
BC023105
Apol9a
Bcl6
Gm6484
Mafb
-7 -6 -5 -4 -3 -2 -1 0 1 2 3 4 5 6 7 8 9

Supplementary Figure S5

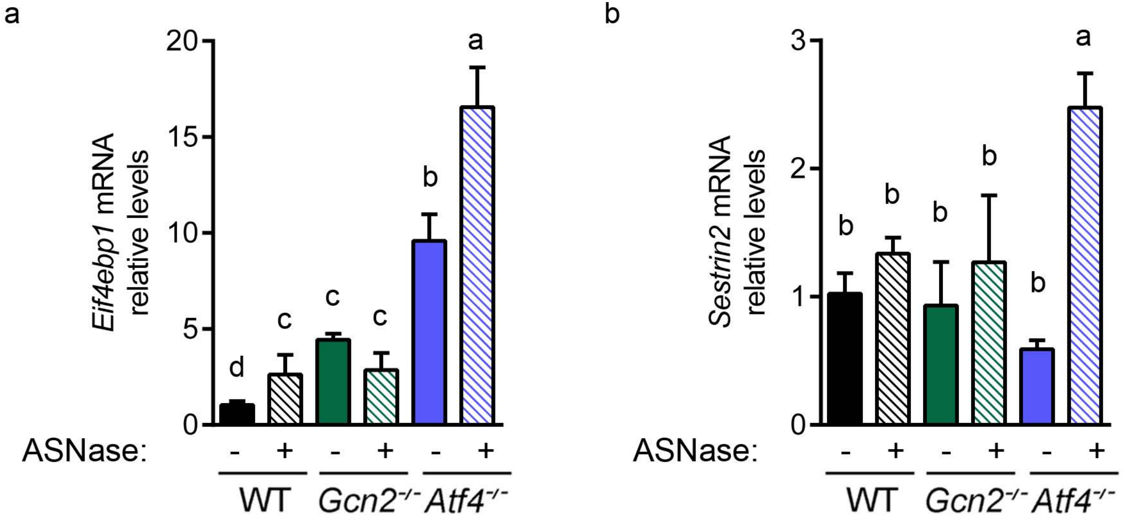

Supplementary Figure 6

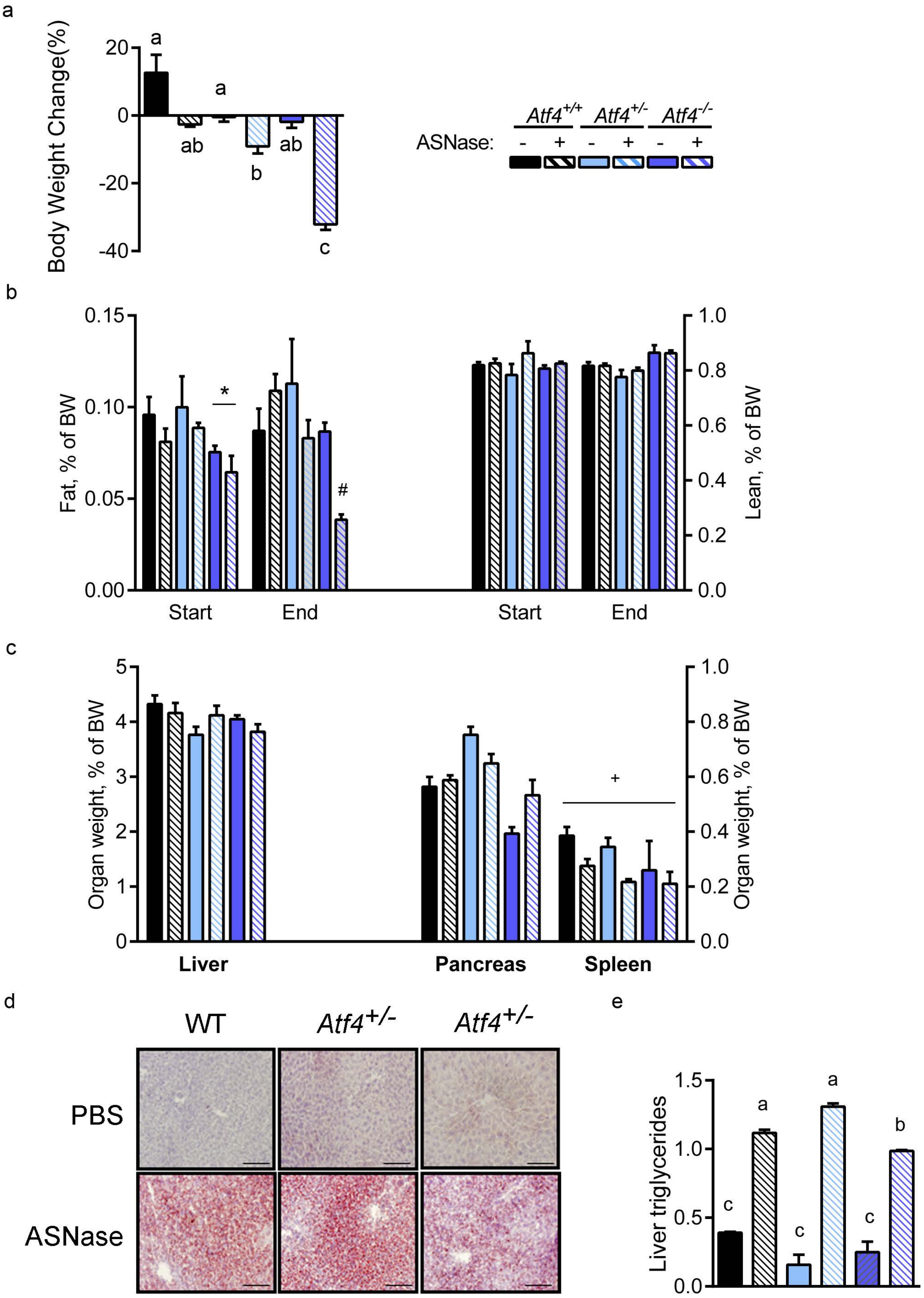

Supplemental Figure 7. Full immunoblots of representative images.

Figure 3. Phosphor-Ser51 eIF2 alpha

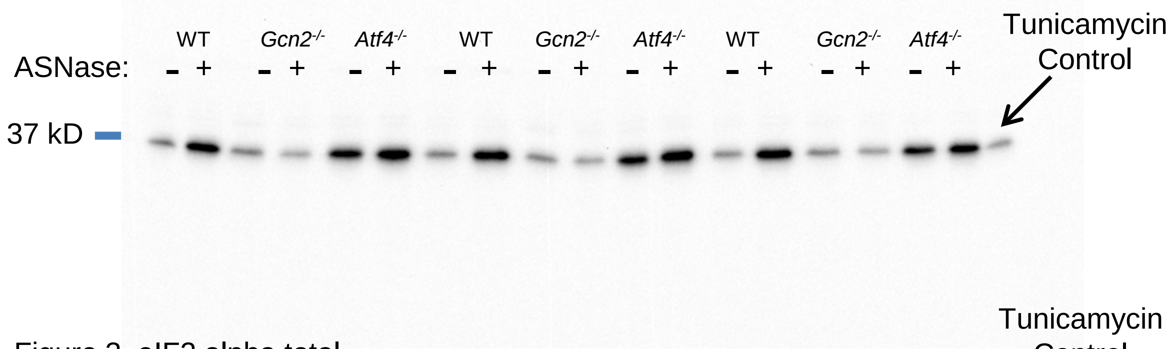

Figure 3. eIF2 alpha total

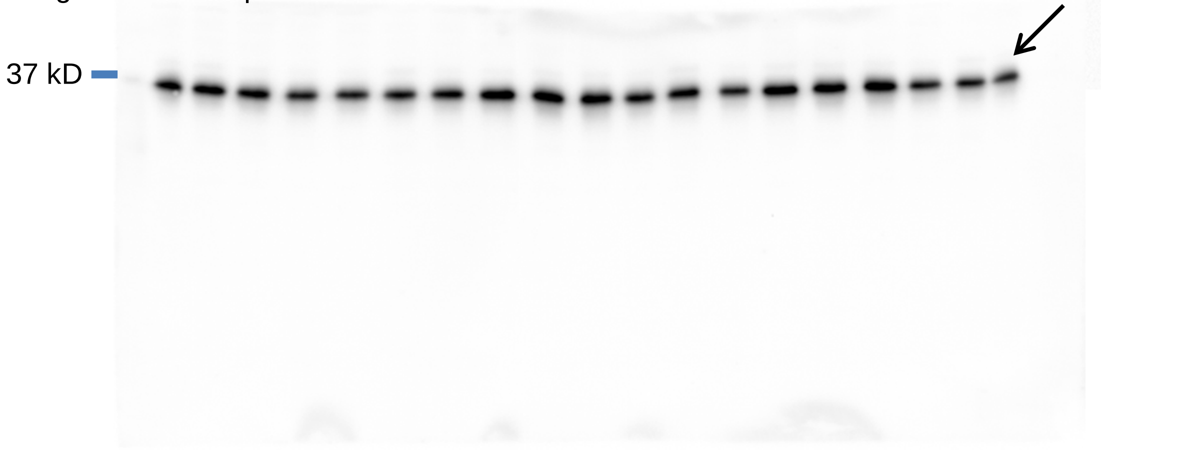

Supplemental Figure 7. Full immunoblots of representative images.

Figure 4. Phosphor-Thr980 PERK

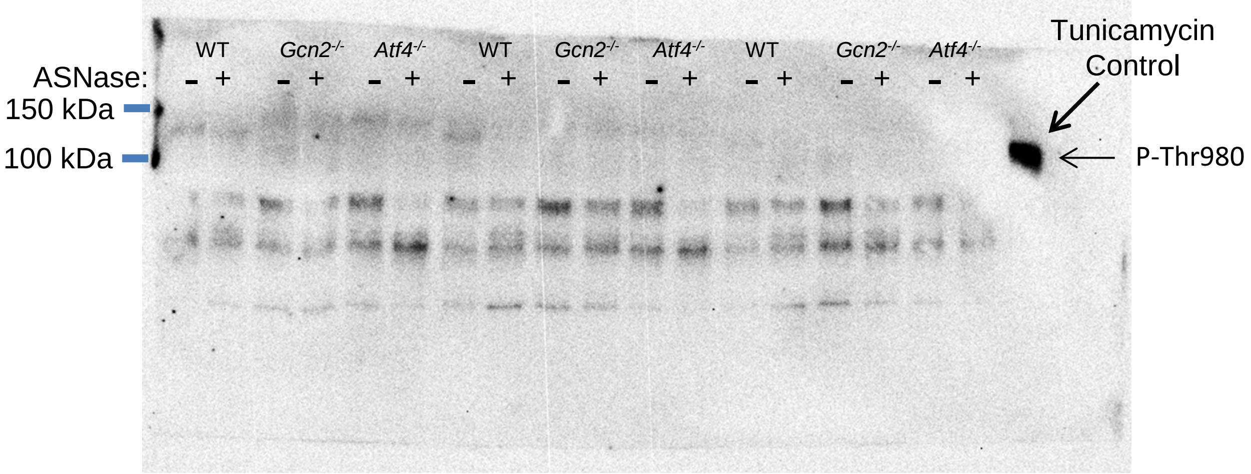

Figure 4. Total PERK

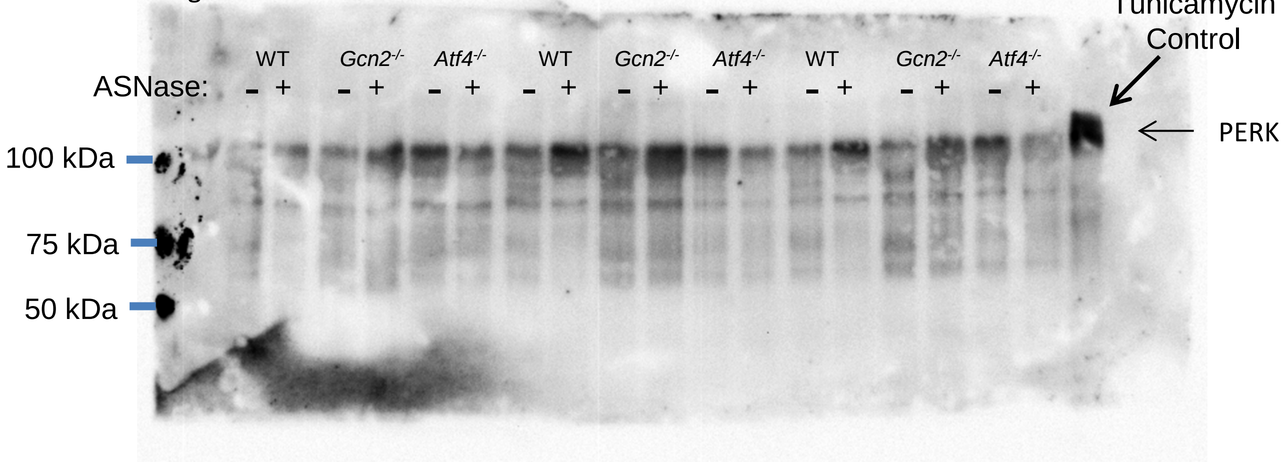

Figure 4. CHOP

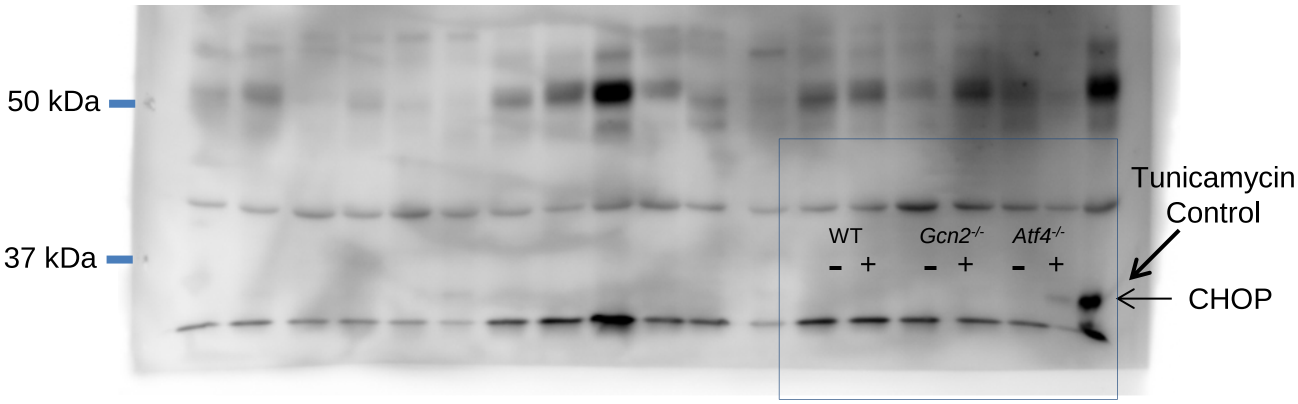

Figure 4. GAPDH

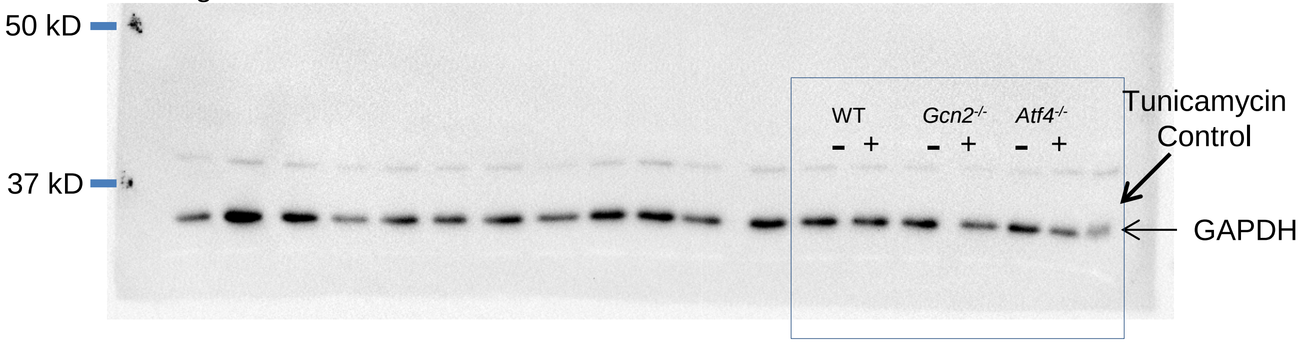

Supplemental Figure 7. Full immunoblots of representative images.

Figure 5. Phosphor-Thr389 S6K1

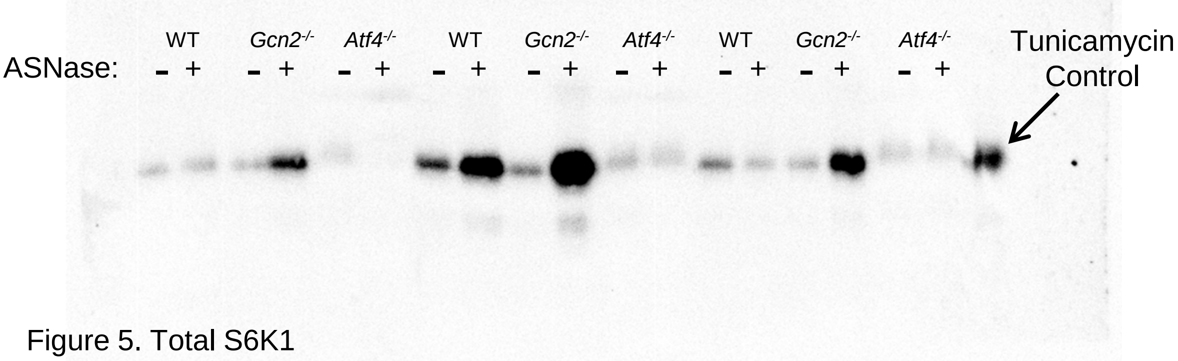

Figure 5. Total S6K1

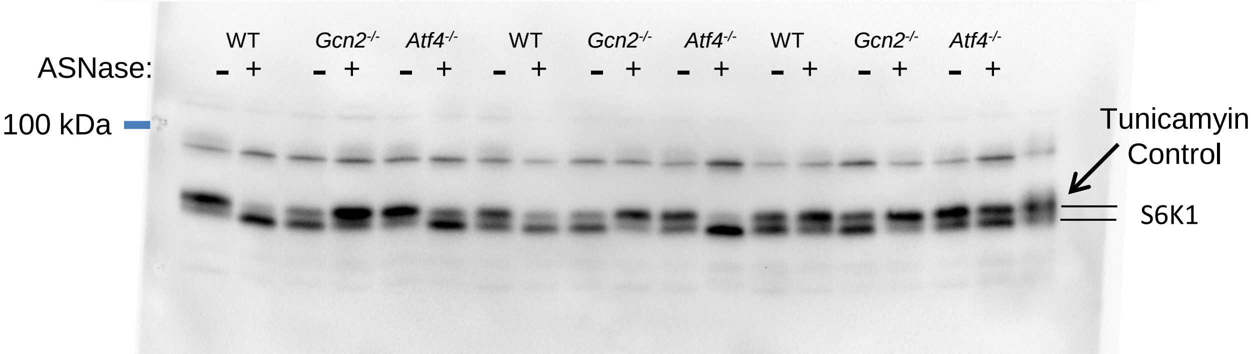

Figure 5. 4E-BP1

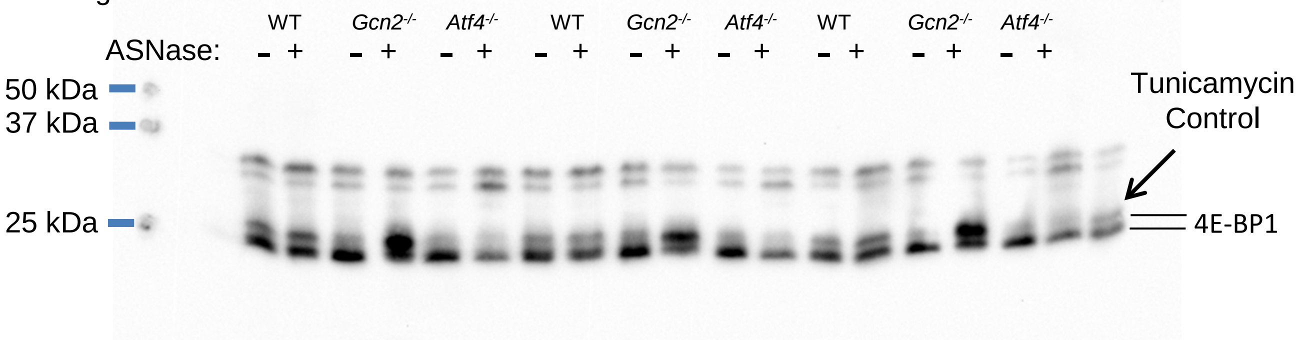

Figure 5. GAPDH

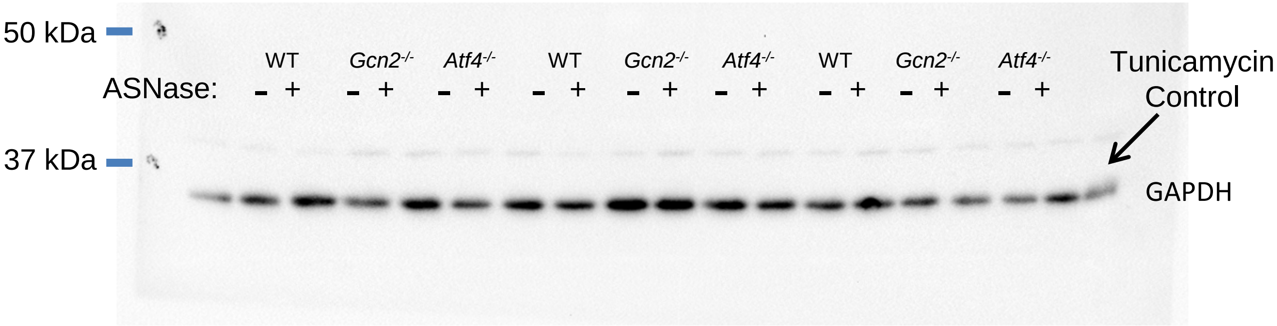

Supplemental Figure 7. Full immunoblots of representative images.

Figure 5. Phosphor-Thr308 Akt

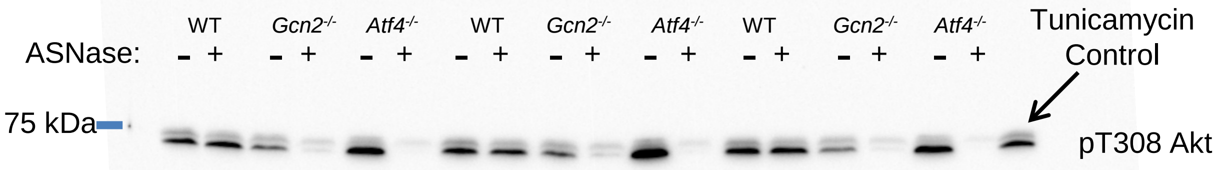

Figure 5. Akt total

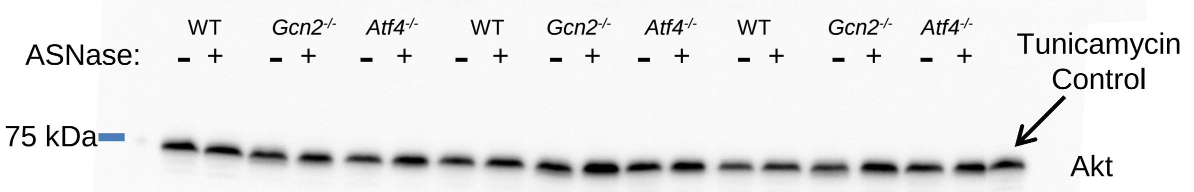

Figure 5. Sestrin2

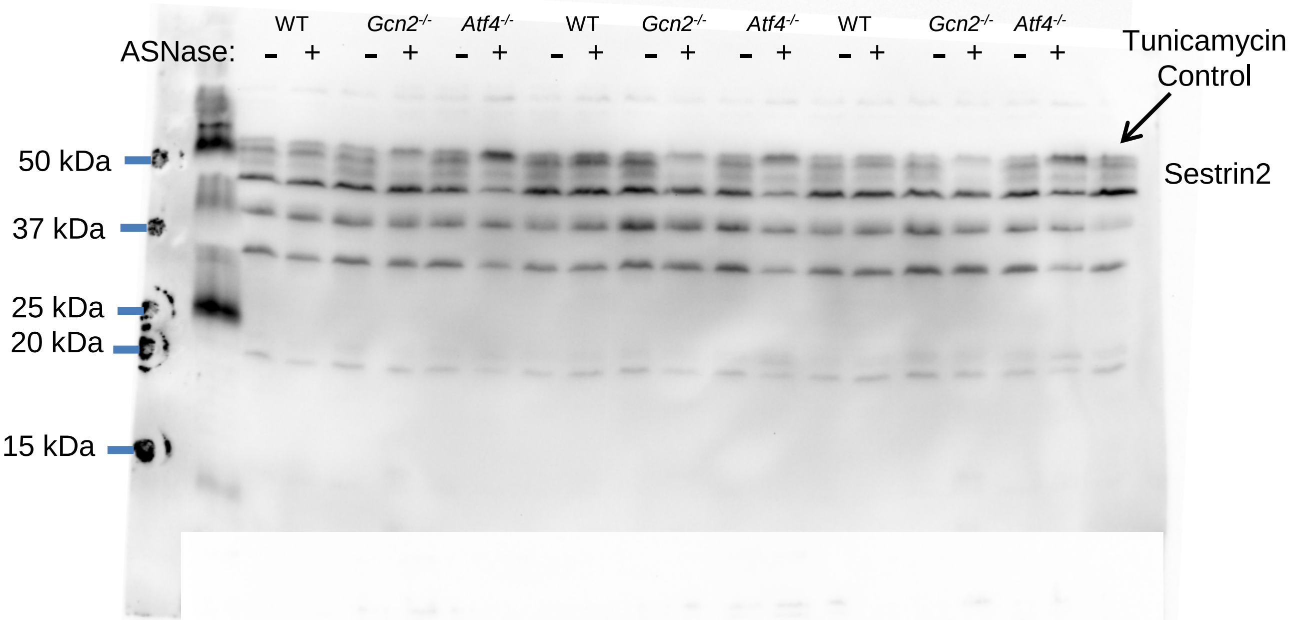

Figure 5. Sestrin2 condensed

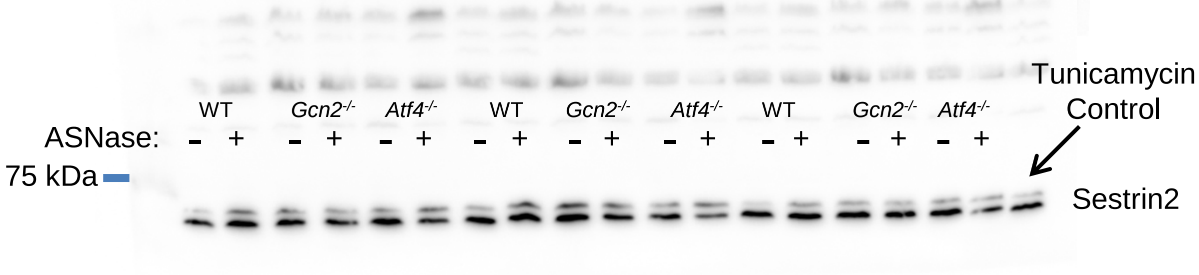

Supplemental Figure 7. Full immunoblots of representative images.

Figure 6. Phosphor-Ser51 eIF2 alpha

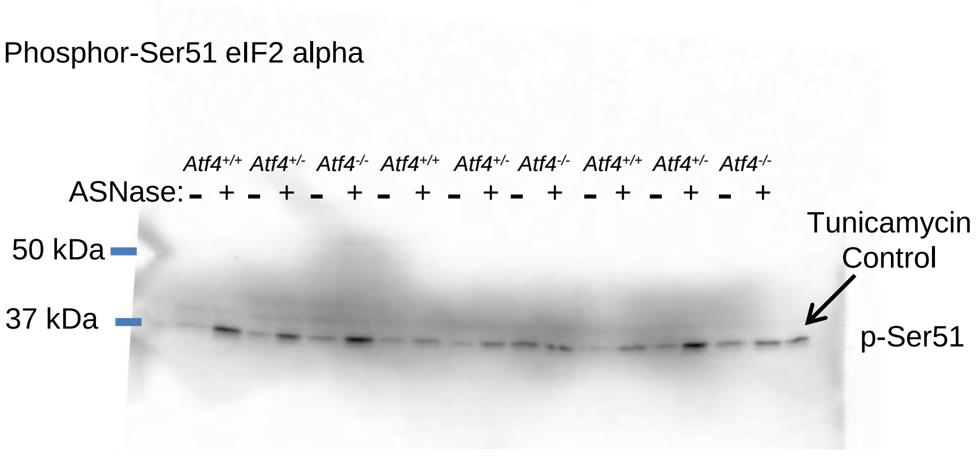

Figure 6. eIF2 alpha total

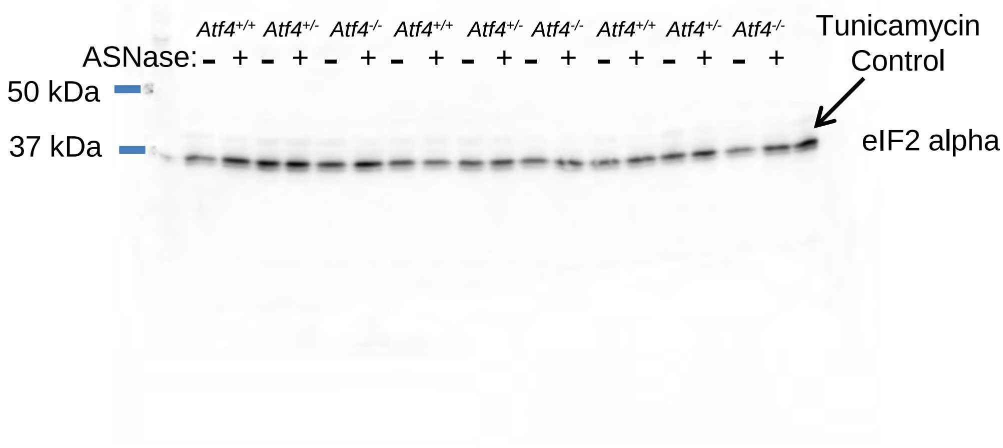

Legend for Supplementary Tables

Supplementary Table S1. Determination of RNA-seq data mapping quality. The table represents mapped percentage of sequenced samples (n=3) of WT, *Gcn2*$^{-/-}$ and *Atf4*$^{-/-}$ mice after being aligned to the mouse genome.

Supplementary Table S2. Gene expression profiling of livers from WT, *Gcn2*$^{-/-}$ and *Atf4*$^{-/-}$ mice treated with PBS or ASNase. Genes altered basally (PBS-injected) divided into three categories: unique to *Gcn2* deletion (A), unique to *Atf4* deletion (C) or common to deletion of either (B). Genes altered by ASNase divided into the following categories: unique to WT (D), unique to *Gcn2*$^{-/-}$ (E); common to *Gcn2*$^{-/-}$ or *Atf4*$^{-/-}$ (F), unique to *Atf4*$^{-/-}$ (G), common to WT and *Gcn2*$^{-/-}$ (H), common to all strains (I), common to WT and *Atf4*$^{-/-}$ (J). Data represent n=3 per group. All differentially expressed genes shown were statistically significant (q value or FDR < 0.1).

Supplementary Table S3. Lists of genes assigned to biological pathways by Ingenuity Pathway Analysis software for Venny categories A-G as depicted in Figure 2B and 2D.

Supporting Table S1.

Sample Name	Number of Reads	Number of Mapped Reads	% of mapping	Median FPKM (isoforms)	Mean FPKM (isoforms)	Num of transcripts (FPKM > 1)	Num of transcripts (FPKM > 0.1)	Num of Genes (FPKM > 1)	Num of Genes (FPKM > 0.1)
WT_PBS_0	32819600	28661655	87.3309	0.267	21.5	12256	16507	10741	13853
WT_PBS_1	34805250	31198176	89.6364	0.324	23.1	12394	16920	10927	14253
WT_PBS_2	38876037	35118530	90.3346	0.428	20.8	13007	17393	11380	14437
WT_ASN_0	36113053	31702489	87.7868	0.354	21	12582	17163	10992	14344
WT_ASN_1	41524626	38190405	91.9705	0.558	20	13541	18125	11828	15099
WT_ASN_2	39539927	34719840	87.8096	0.29	19.7	12392	16788	10828	14032
GCN2_KO_PBS_0	34673607	30427066	87.7528	0.259	19.8	12156	16443	10669	13828
GCN2_KO_PBS_1	36683796	33550168	91.4577	0.214	22	11868	16205	10433	13555
GCN2_KO_PBS_2	38346900	34763978	90.6566	0.257	22.7	12170	16480	10671	13778
GCN2_KO_ASN_0	32430908	29692330	91.5557	0.302	23.8	12590	16643	10980	13851
GCN2_KO_ASN_1	33266075	29751635	89.4354	0.19	25.8	11843	15976	10442	13393
GCN2_KO_ASN_2	35564155	32616474	91.7117	0.256	21.1	12256	16403	10727	13680
ATF4_KO_PBS_0	41066020	36904486	89.8662	0.339	20.6	12668	16976	11082	14092
ATF4_KO_PBS_1	38721303	33159631	85.6367	0.309	20.8	12512	16839	10903	14015
ATF4_KO_PBS_2	39172739	34207403	87.3245	0.269	20.9	12274	16631	10755	13853
ATF4_KO_ASN_0	36547497	33476011	91.5959	0.202	21.6	11728	16103	10303	13458
ATF4_KO_ASN_1	43122273	38467984	89.2068	0.354	20.1	12720	17093	10967	14165
ATF4_KO_ASN_2	43068901	40107544	93.1241	0.283	19.8	12293	16774	10717	13923
